# Personalization of biomechanical simulations of the left ventricle by in-vivo cardiac DTI data: Impact of fiber interpolation methods

**DOI:** 10.3389/fphys.2022.1042537

**Published:** 2022-11-28

**Authors:** Johanna Stimm, David A. Nordsletten, Javiera Jilberto, Renee Miller, Ezgi Berberoğlu, Sebastian Kozerke, Christian T. Stoeck

**Affiliations:** ^1^ Institute for Biomedical Engineering, University and ETH Zurich, Zurich, Switzerland; ^2^ Department of Biomedical Engineering and Cardiac Surgery, University of Michigan, Ann Arbor, MI, United States; ^3^ School of Biomedical Engineering and Imaging Sciences, King’s College London, London, United Kingdom; ^4^ Division of Surgical Research, University Hospital Zurich, University Zurich, Zurich, Switzerland

**Keywords:** *in vivo* cDTI, patient-specific modelling, cardiac microstructure, fiber interpolation, cardiac simualtion, *in vivo* microstructure, personalized modelling

## Abstract

Simulations of cardiac electrophysiology and mechanics have been reported to be sensitive to the microstructural anisotropy of the myocardium. Consequently, a personalized representation of cardiac microstructure is a crucial component of accurate, personalized cardiac biomechanical models. *In-vivo* cardiac Diffusion Tensor Imaging (cDTI) is a non-invasive magnetic resonance imaging technique capable of probing the heart’s microstructure. Being a rather novel technique, issues such as low resolution, signal-to noise ratio, and spatial coverage are currently limiting factors. We outline four interpolation techniques with varying degrees of data fidelity, different amounts of smoothing strength, and varying representation error to bridge the gap between the sparse *in-vivo* data and the model, requiring a 3D representation of microstructure across the myocardium. We provide a workflow to incorporate *in-vivo* myofiber orientation into a left ventricular model and demonstrate that personalized modelling based on fiber orientations from *in-vivo* cDTI data is feasible. The interpolation error is correlated with a trend in personalized parameters and simulated physiological parameters, strains, and ventricular twist. This trend in simulation results is consistent across material parameter settings and therefore corresponds to a bias introduced by the interpolation method. This study suggests that using a tensor interpolation approach to personalize microstructure with *in-vivo* cDTI data, reduces the fiber uncertainty and thereby the bias in the simulation results.

## 1 Introduction

Despite recent improvements in medical care, cardiovascular disease remains the leading cause of death worldwide (World Health Organization, 2021). Advances in early detection and prediction of disease progression after the first cardiac event are essential to prevent irreversible damage and manifestation of secondary disease. To this end, studies on disease development with patient-specific models are a great opportunity to better understand the underlying mechanisms of pathology and can advance early diagnostics and patient-specific treatments (Kayvanpour et al., 2015).

The heart is a multi-physics, multi-scale system with a complex tissue structure. The myocytes are grouped in myocyte aggregates arranged in a double helical pattern on the organ scale, while forming a branching and inter-connecting network on a smaller scale (Streeter et al., 1969; Gilbert et al., 2007). Myocyte aggregates are arranged in myolaminae (LeGrice et al., 1997; Gilbert et al., 2007; Lunkenheimer and Niederer, 2012), a second component of the local myocyte orientation. This characteristic arrangement is represented by the fiber-sheet structure in biomechanical models.

The cardiac microstructure is an essential component of computational biomechanical models. Myocyte orientation determines the direction of active tension during contraction and the fiber-sheet structure is responsible for the anisotropy of the tissue, influencing its passive response. Further, cardiac microstructure and microstructure dynamics were shown to be altered in pathology, such as hypertrophic and dilated cardiomyopathy (Ferreira et al., 2014; von Deuster et al., 2016a), and aortic stenosis (Gotschy et al., 2021). Due to the involvement of microstructure in cardiac disease and associated remodelling, a patient-specific representation is essential in personalized cardiac models.

However, most state-of-the-art models rely on a generic representation of microstructure, either defined by rule-based methods, statistical atlases, or single *ex-vivo* cardiac Diffusion Tensor Imaging (cDTI) data sets morphed onto a patient-specific geometry. Rule-based methods typically represent the fiber orientation as a function of transmural position prescribing generic values of the characteristic helix and transverse angles at the endo- and epicaridal boundaries (Beyar and Sideman, 1984; Potse et al., 2006). Extensions with spatial variations have been proposed (Karadag et al., 2011). A rule-based method to model cardiac microstructure in cardiac biventricular geometries including the outflow tract has been introduced by Doste et al. (2019). A well established approach to prescribe a transmurally varying rule-based fiber orientation is based on physiological coordinates following the heart shape and is obtained by solving a Laplace problem with Dirichlet boundary conditions (Bayer et al., 2012; Rossi et al., 2014; Wong and Kuhl, 2014; Doste et al., 2019). Statistical atlases have been extracted from *ex-vivo* cDTI data of multiple hearts (Peyrat et al., 2007; Lombaert et al., 2012b,a; Piuze et al., 2013; Lekadir et al., 2014; Zhang and Wei, 2017; Mojica et al., 2020) or *in-vivo* cDTI upon interpolation by Toussaint et al. (2010, 2013). These population averaged fiber fields are then morphed onto individual geometries of the heart.

The sensitivity of simulation results (such as torsion, stress, and strain distributions) to microstructure has been investigated in previous studies, revealing an essential influence of fiber orientation suggesting the need for individualized microstructure in patient-specific simulations (Geerts et al., 2003; Wang et al., 2013; Palit et al., 2015; Nikou et al., 2016b; Pluijmert et al., 2017; Gil et al., 2019; Campos et al., 2020; Guan et al., 2020; Barbarotta et al., 2021; Rodríguez-Padilla et al., 2022). Sensitivity studies using a rule-based fiber orientation with varying helix and transverse angles have revealed changes in the distribution of myofiber stress and shortening of up to 9 percentage points resulting from a difference in fiber direction of 8° (Geerts et al., 2003). Similarly, Pluijmert et al. (2017) showed that a variation in fiber orientation of 8°, that was generated with an automatic fiber reorientation method, led to a change in local myofiber work and pump work of 11–19%. In a diastolic left-ventricular model, Wang et al. (2013) showed that changing the endocardial helix angle influences the transmural distribution of fiber stress. A variation of the endocardial helix angle of 60° by +10°/−10° resulted in a change in fiber stress of approximately 31%/26% at a mid-ventricular, mid-mural location. This sensitivity of the fiber stress to the microstrucure orientation was likewise demonstrated for fiber and sheet orientation in a study by Nikou et al. (2016b), extending previous investigations from evaluations within one short-axis region to multiple longitudinal slices. In a diastolic bi-ventricular model, the variation of the fiber orientation, was investigated by Palit et al. (2015), confirming the sensitivity of fiber stress and strain distribution depending on fiber orientation and showing an effect on stiffness. The effect of microstructure on torsion was demonstrated by Campos et al. (2020) in a sensitivity analysis of the left ventricle during the full cardiac cycle. Barbarotta et al., 2021 revealed that the transverse angle has a higher influence on the end-systolic strains than the helix angle with shear strains being the most sensitive strain components. Further, they found a higher sensitivity of cardiac strains to microstructure than to variation in geometry, suggesting that the lack of individual microstructure in patient-specific models might hamper precise systolic strain estimation. Rodríguez-Cantano et al. (2019) and Eriksson et al. (2013) identified a high sensitivity of cardiac simulation outputs to local variation in fiber orientation. The differences between homogeneous *versus* heterogeneous fiber fields was further investigated in a study by Gil et al. (2019). They showed that torsion and long-axis shortening were closer to healthy data in a model with fiber architecture derived from *ex-vivo* cDTI data compared to two models with rule-based fiber representations adapted to data from histology. This influence of realistic heterogeneous microstructure is confirmed in a study by Guan et al. (2020), comparing three models with fiber fields generated with different approaches from the same *ex-vivo* cDTI data. The more realistic fibers obtained by directly mapping the *ex-vivo* cDTI data onto the geometry resulted in physiological values of cardiac output, higher ejection fraction, and larger apical twist. Additional active contraction in cross-fiber directions was needed for the sector-wise and a global rule-based representation, to achieve physiological behaviour. In an *ex-vivo* analysis of the myocardial tissue, Rodríguez-Padilla et al. (2022) found a bi-layer structure in the septum, with two different primary directions of fiber orientation and showed the influence of this local feature on the stress distribution. These sensitivity studies suggest that patient-specific, locally heterogeneous microstructure is important for patient-specific modelling.

Recent advances in *in-vivo* cDTI enable non-destructive measurements of *in-vivo* microstructural orientations (Nguyen et al., 2016; Stoeck et al., 2016; Scott et al., 2018; Stoeck et al., 2018; Moulin et al., 2020), providing a way to obtain patient-specific structural information. However, cardiac motion influences the diffusion signal and therefore *in-vivo* cDTI is challenging. Two magnetic resonance sequences are typically used: 1) motion compensated spin echo (SE) sequences (Welsh et al., 2015; Stoeck et al., 2016) or 2) stimulated echo sequences (STEAM) (Tseng et al., 1999; Dou et al., 2002; Nielles-Vallespin et al., 2013; Stoeck et al., 2014). STEAM sequences encode diffusion over two consecutive heart beats, requiring that the heart is in the same position and motion state over two cardiac cycles. This leads to long scan times and necessitates good patient compliance due to repetitive breath holding. SE sequences allow for acquisitions in one heartbeat and thus can be performed during free breathing. However, high gradient strength is needed, requiring high-performance hardware. A detailed comparison of both approaches has been performed by von Deuster et al. (2016b); Scott et al. (2018). *In-vivo* cDTI currently suffers from low spatial resolution, low signal-to-noise ratio, and reduced spatial coverage with a limited number of short-axis slices. In-plane resolutions of 1.6 × 1.6 mm^2^ (Moulin et al., 2020) or 1.8 × 1.8 mm^2^ (Gorodezky et al., 2019) have been reported *in-vivo* when using parallel imaging or multi-shot acquisition schemes, that allow to shorten the read-out duration. Patient compliance and long scan times restrict the spatial coverage in clinical *in-vivo* studies. Typically, one to three short-axis slices are acquired (Nielles-Vallespin et al., 2013; Khalique et al., 2020, 2018; Gotschy et al., 2021). Increasing spatial coverage in a clinical setting is ongoing research (Nguyen et al., 2021). Therefore, data pre-processing and interpolation techniques are necessary to use this sparse data to personalize 3D cardiac models.

In this work, we address the gap between available *in-vivo* micro-structural data and the requirements for biomechanical modelling. We personalize a left-ventricular model to a porcine heart based on MRI data with subject-specific cardiac microstructure from *in-vivo* cDTI data. To this end, we employ four interpolation techniques with varying degrees of freedom to map the sparse *in-vivo* data to the model: one tensor interpolation approach (Toussaint et al., 2013), two parametric, low-rank models extracted from *ex-vivo* data (Stimm et al., 2021), and a rule-based method (Bayer et al., 2012) adapted to the data. The methods have been previously compared with respect to interpolation performance (Stimm et al., 2022). The tensor interpolation approach resulted in the lowest interpolation errors followed by the low-rank models (PGD and POD) and the rule-based method. An *ex-vivo* experiment with synthetically down-sampled data suggested that interpolation benefits more from an increase in in-plane resolution from 2.5*mm* ^2^ to 1.5 mm^2^ of the measured input data compared to improvements of signal-to-noise ratio and number of input slices. Here, we study the sensitivity of the simulation output to the interpolation techniques with varying smoothness and fidelity.

## 2 Methods

### 2.1 Data acquisition and processing

#### 2.1.1 Imaging

One healthy porcine heart was imaged on a clinical 1.5 TMR system (Achieva, Philips Healthcare, Best, Netherlands) with a 32-channel cardiac coil and gradient system parameters: gradient strength: 80 mT/m per physical axis; slew rate: 100 T/m/s. Data was collected from previous studies (Stoeck et al., 2021; Stimm et al., 2022). Experimental procedures were approved by the Cantonal Veterinary Office (Zurich, Switzerland) under licenses ZH072/16 and ZH 152/2013. Image acquisition was performed during ventilated breathing. Multi-slice, short-axis cine imaging (cine) and cDTI were performed with the following parameters: cine: 1.8 × 1.8 mm^2^ spatial in-plane resolution, 8 *mm* slice thickness, 25 heart phases, TE/TR 1.5 ms/3 ms, 45°flip angle; cDTI: 2.0 mm^2^ × 2.0 mm^2^ spatial in-plane resolution, zero-filled to 1.3 × 1.3 *mm* ^2^, 8 *mm* slice thickness, 3/3/12 encoding directions at b = 100/200/450 s/*mm* ^2^, TR/TE 5R-R intervals/81 ms, and eight signal averages. cDTI was acquired with a second-order motion-compensated spin-echo sequence (Welsh et al., 2015; Stoeck et al., 2016) and triggered to 65% of peak contraction (Stoeck et al., 2016, 2018). The field-of-view was limited in the phase encoding direction using non-coplanar excitation (Wilm et al., 2009). Ten consecutive slices were acquired in an interleaved fashion within two scans with five slices and a slice gap of 8 mm each.

#### 2.1.2 Cine-based motion tracking

The end-systolic frame of the cine images was manually segmented using MeVisLab (MeVis Medical Solutions, Bremen, Germany). Consecutive motion tracking of the mesh over the cardiac cycle was performed. To this end, displacement fields of all imaged time-frames were estimated with a FEM-based image registration approach (Genet et al., 2018; Berberoğlu et al., 2021). Registration was performed with a dilated geometry to enable feature tracking at the endo- and epicardial boundaries. Dilation to obtain a boundary layer of 2–3 mm around the ventricle was performed using GMSH (Geuzaine and Remacle, 2009).

**FIGURE 1:**
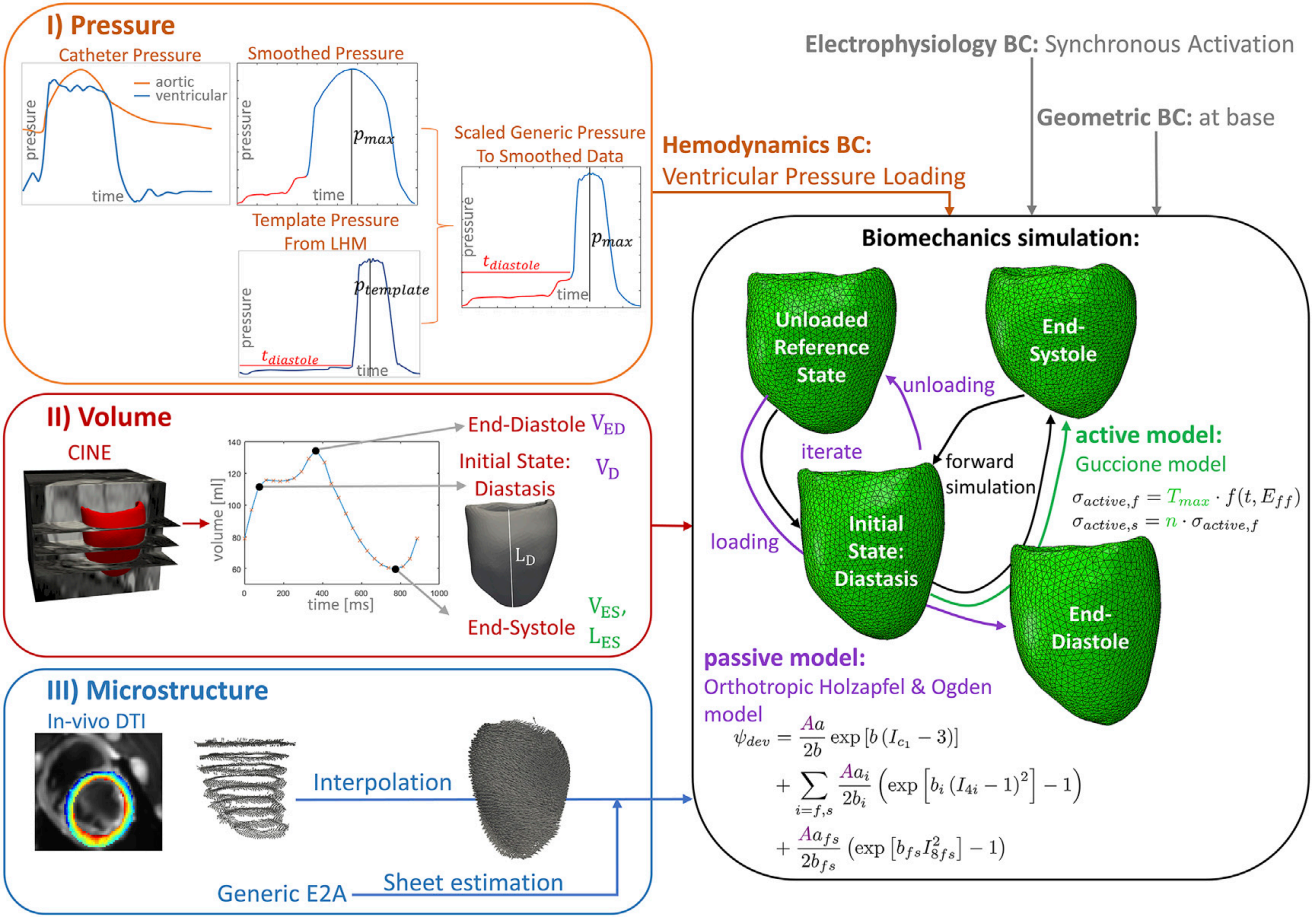
Workflow of the data assimilation and personalization process for a left-ventricular biomechanics model of one porcine heart. I) (orange box) Ventricular and aortic catheter pressure measurements are combined and smoothed. The diastolic pressure trace and the maximal systolic pressure value are extracted to personalize a template pressure curve extracted from the underlying Living Heart Model (LHM), while keeping the duration of diastole, systolic pressure increase, and pressure decrease of the template pressure curve constant. This pressure trace provides the hemodynamic boundary condition for the simulation (orange input). II) (red box) The volume over the cardiac cycle is extracted from cine images using tracking. The mesh extracted in diastasis is used as initial state for the simulation. End-diastolic and diastatic volumes are used to adapt the passive model parameters by scaling the parameter A (purple) and to estimate an unloaded reference state. The end-systolic frame is used to adapt the active model (green). The end-systolic volume is used to fit the maximal active tension parameter *T*_max_, the left-ventricular length of this frame is used to adapt the parameter *n*, scaling the active stress contribution in sheet direction. III) (blue box) The predominant aggregated myocyte orientation is measured with *in-vivo* cDTI in eight short-axis slices. Four interpolation techniques are applied to obtain a 3D fiber field on the 3D mesh. The sheet direction is estimated to reach a diastolic E2A angle of 13°(Nielles-Vallespin et al., 2017). The biomechanics model uses the LHM implementation (Baillargeon et al., 2014) of the passive orthotropic Holzapfel and Ogden (Holzapfel and Ogden, 2009) material model and the active Guccione model (Guccione et al., 1995). The reference geometry is estimated by unloading the initial state using a suction problem and subsequent loading to end-diastole. This procedure (purple arrows) is repeated while iterating over the passive material scaling (A). The volume after loading is compared to the data at the loading condition of the initial diastatic pressure and at end-diastole. The sum of these volume errors serves as objective function to identify the unloaded reference and passive scaling parameter estimates. The simulation over the cardiac cycle is performed starting from the unloaded reference (black arrows).

The volume of the deformed left-ventricular (LV) geometries was estimated by partitioning the endocardial surface, connecting the surface nodes to the center of the basal plane, and calculating the sum over all resulting tetrahedral elements using VTK (Visulatization Toolkit, Kitware, Inc.). The volume was evaluated at end-diastole (*V*_*ED*_), diastasis (*V*_*D*_), and end-systole (*V*_*ES*_). The mesh was extracted at diastasis and re-meshed with 160,000 elements using GMSH to provide the initial configuration of the simulation study. As depicted in the red box in Figure 1 II), the volumes *V*_*ED*_, *V*_*D*_, *V*_*ES*_, and the left-ventricular length in diastasis *L*_*D*_ and end-systole *L*_*ES*_ served as inputs for model personalization (Section 2.3.4). The length was calculated as the Euclidean distance between the apex and the center of the base. *L*_*ES*_ corresponds to maximal left-ventricular shortening.

#### 2.1.3 Pressure measurements

The left-ventricular and aortic pressure were measured prior to MRI in the surgical theater with an open pig tail catheter placed in the LV cavity and aorta, respectively, and connected to an external pressure sensor. The reference pressure was calibrated to the ambient pressure. The pressure was measured over 30 cardiac cycles and retrospectively averaged. Post-processing is depicted in Figure 1 Box I. The averaged pressure traces were smoothed to damp oscillations. To correct for uncertainties in the LV pressure due to strong oscillations introduced by the valve motion, the aortic and ventricular pressure curves were merged. While the aortic valve was open, the aortic pressure values were considered to coincide with the ventricular pressure, neglecting pressure gradients. To enable the use of previously calibrated active model parameters (Walker et al., 2005; Baillargeon et al., 2014), depending on the timing of rapid pressure increase and systolic length, the pressure curve was combined with a generic pressure curve. The diastolic pressure trace and the value of the peak ventricular pressure *p*_max_ were obtained from the pressure data and used to adapt a generic pressure curve, subsequently applied as the hemodynamic loading condition as outlined in Section 2.3.3.

#### 2.1.4 *In vivo* cardiac diffusion tensor data processing

*In-vivo* data processing was performed as previously reported (Stimm et al., 2022). To summarize, residual spatial mismatch of acquisitions with different b-values and diffusion encoding directions due to ventilated breathing were compensated for by non-rigid registration (Vishnevskiy et al., 2017). Then, the diffusion tensor was solved for on a pixel-by-pixel basis in Matlab (MathWorks, Inc.) inverting the system of linear equations using the Moore-Penrose pseudo-inverse. The 2D short-axis images were manually segmented between the apex and the base, resulting in nine segmented short-axis slices. A corresponding 3D geometry was estimated using a marching cube algorithm in MeVisLab (MeVis Medical Solutions, Bremen, Germany). Physiological, shape-adapted coordinates with global transmural, circumferential, and longitudinal coordinates and local coordinate axes were calculated (Bayer et al., 2005, 2012; Paun et al., 2017; Doste et al., 2019). To this end, a total of four heat transfer problems with Dirichlet boundary conditions were solved in Abaqus 2020 (Simulia, Dassault Systèmes). A steady state analysis was performed such that the problem is equivalent to the commonly applied Laplace equation (Bayer et al., 2018). To obtain the transmural coordinates, the boundary conditions were defined at the endocardial and the epicardial surfaces (endo: t = 0, epi: t = 1). At the apex a transmural cylinder with a diameter of 2 mm was defined. To obtain the longitudinal coordinates, a second heat transfer problem was solved between the basal plane (base: l = 1) and the apex cylinder (apex: l = 0). Then, two boundary value problems were solved with boundary conditions at the plane through the anterior intersection of left and right ventricle and the apex, splitting the ventricle into two-halves. For these two heat flux simulations, the transmural cylinder at the apex was defined as an insulating material. The solution on both halves of the LV were combined to obtain the circumferential coordinates. Within the cylinder at the apex, cylinder coordinates were used to define the local circumferential coordinates. The vectors of all resulting heat flux fields in transmural, longitudinal, and circumferential direction were re-orthogonalized and correspond to the local coordinate axes. The global coordinates were obtained from streamline tracking and normalization to the maximal streamline length, similar to the normalization by Paun et al. (2017). These coordinates were calculated both on the geometries obtained from the cDTI data and the initial geometry used for simulation from the cine data, enabling to transfer the measured microstructure from cDTI data onto the mesh used for simulation.

The diffusion tensor’s first eigenvector, corresponding to the average cardiomyocyte long-axis orientation was calculated for each voxel within the myocardium. To overcome the sign invariance, it was ensured that the eigenvectors had a positive circumferential orientation. The average cardiomyocyte long-axis orientation corresponds to the primary symmetry-axis, considered in the mechanical material model and termed fiber direction in the following. Helix and transverse angles were calculated with respect to the local coordinate system. The helix angle was defined as the angle between the projection of the fiber direction onto the longitudinal-circumferential plane and the circumferential axis (Scollan et al., 1998; Stoeck et al., 2018). If the fiber direction and the longitudinal axis point into the same half-space, the value is positive, otherwise it is negative. The transverse angle was defined as the angle between the local circumferential axis and the projection of the fiber direction onto the local transmural-circumferential plane (Scollan et al., 1998). It is positive if the projection and the transmural axis point into the same half-space.

### 2.2 Interpolation of *in vivo* average cardiomyocyte long-axis orientation

We compared four interpolation methods to interpolate sparse *in-vivo* cDTI data onto a fine 3D mesh used for cardiac LV simulations: 1) one tensor interpolation approach using the method proposed by (Toussaint et al., 2010, 2013) with shape-adapted, heat flux coordinates (Section 2.1.4) (HFC), 2) and 3) two low-rank models, extracted from *ex-vivo* data in a previous work (Stimm et al., 2021) with adjustable parameters based on a Proper Generalized Decomposition and Proper Orthogonal Decomposition, respectively (PGD and POD), and 4) one rule-based method, based on the work of Bayer et al. (2018), which only considers the LV and adapts the parameters to the data (RBM). All interpolation methods were based on the physiological coordinates outlined in Section 2.1.4. The interpolation error was approximated, as previously prescribed in (Stimm et al., 2022), by first excluding one mid-ventricular short axis slice from the cDTI data set, then interpolating the remaining data onto the coordinates of the excluded mid-ventricular slice. Finally, we compared the interpolated fiber vectors to the original cDTI’s first eigenvector within this mid-ventricular slice and evaluated the absolute angular difference.

#### 2.2.1 Tensor interpolation (HFC)

The tensor interpolation approach is based on the work of Toussaint et al. (2010, 2013). The cDTI tensors were transformed into the local shape-adapted coordinate system. The interpolated diffusion tensor *D*_*approx,j*_ at location ${\vec{x}}_{j}$ is a weighted average of the input tensors in a log-Euclidean framework:

$$D_{\mathrm{approx,j}}=\exp\left(\frac{\sum_{i=1}^{N}w_{i,j}\log\left(D_{i}\right)}{\sum_{i=1}^{N}w_{i,j}}\right),$$ (1)

with input tensors *D*_*i*_ at all *N* data points with positions ${\vec{x}}_{i}$ , and weights *w*_*i*,*j*_. To obtain the weights, an anisotropic Gaussian kernel function was defined by a pre-computed weighting matrix $H\in R^{3x3}$ , that depends on the sparsity, resolution, and signal-to-noise ratio for the available data. This matrix was optimized for the underlying setting of the input data based on *ex-vivo* data in a previous study (Stimm et al., 2022) using a least squares approach (Toussaint et al., 2013). The resulting weights for each target position ${\vec{x}}_{j}$ depend on the distance $dX_{i,j}⃗={\vec{x}}_{j}-{\vec{x}}_{i}$ to the input data position ${\vec{x}}_{i}$ :

$$w_{i,j}=\frac{\exp\left(-dX_{i,j}^{T}⃗H^{-2}dX_{i,j}⃗\right)}{\sqrt{2\pi }\cdot \det\left(H\right)}.$$ (2)

The first eigenvector of the interpolated diffusion tensor was calculated to obtain the interpolated fiber directions.

#### 2.2.2 *Ex-vivo* data-driven low-rank models (PGD and POD)

Two data-driven models of the LV fiber orientation have been derived from *ex-vivo* data with an isotropic resolution of 0.5* mm* ^*3*^ from eight porcine hearts in a previous publication (Stimm et al., 2021). Basis functions characterizing the spatial variation of fiber orientation were obtained and combined with a set of weights, that can be used to adapt the models to new data.

To obtain the basis functions for both models, the normalized fiber directions $\vec{f}\left(t,c,l\right)\in R^{3}$ , with $|\vec{f}|=1$ at the position of each voxel center with coordinates (c,t,l), were first projected on the unit vectors in transmural $\left({\vec{e}}_{t}\right)$ , circumferential $\left({\vec{e}}_{c}\right)$ , and longitudinal $\left({\vec{e}}_{l}\right)$ direction, spanning the local coordinate system $\left({\vec{e}}_{t},{\vec{e}}_{c},{\vec{e}}_{l}\right)$ . This resulted in the fiber projections $f_{d}\left(t,c,l\right)=\vec{f}\left(t,c,l\right)\cdot {\vec{e}}_{d}$ , with *d* ∈ {*t*, *c*, *l*}, such that the fiber orientation is represented in the physiological, local coordinate system $\vec{f}\left(t,c,l\right)=\sum_{d\in t,c,l}f_{d}\left(t,c,l\right)\cdot {\vec{e}}_{d}$ . Then for each projection *f*_*t*_ (*t*, *c*, *l*), *f*_*c*_ (*t*, *c*, *l*), and *f*_*l*_ (*t*, *c*, *l*) independently, the data was compressed by applying order-reduction techniques, 1) a Proper Generalized Decomposition combined with a Singular Value Decomposition for the PGD-model, or 2) a Proper Orthogonal Decomposition for the POD-model. Basis functions approximating the main spatial variation of fiber orientation across individual microstructures were obtained. Combining these basis function with adjustable weights resulted in a model for each projection *f*_*d*,*PGD*_ or *f*_*d*,*POD*_, with *d* ∈ {*t*, *c*, *l*}. The final interpolated fiber direction is given by: $f_{\mathrm{interp}}⃗\left(t,c,l\right)=\sum_{d=t,c,l}f_{d,model}\left(t,c,l\right)\cdot e_{d}⃗$ , with *f*_*d*,*model*_ ∈ {*f*_*d*,*PGD*_, *f*_*d*,*POD*_}.

The PGD-model was obtained by first applying a Proper Generalized Decomposition (Chinesta et al., 2011, 2014; Genet et al., 2015) to each heart and fiber projection separately. This results in a low-rank basis decomposition that approximates the data for each individual heart:

$$f_{d,\text{ data one heart}}\left(t,c,l\right)\approx \overset{N_{\text{PGD}}=6}{\underset{m=1}{\sum }}F_{m}\left(t\right)\cdot G_{m}\left(c\right)\cdot H_{m}\left(l\right),$$ (3)

were the *m* ^th^ basis function is a product of three 1D functions (*X*_*m*_(*v*) = *F*_*m*_(*t*), *G*_*m*_(*c*), *H*_*m*_(*l*) with *v* = *t*, *c*, *l*). These 1D functions (*X*_*m*_(*v*)) are discretized with piece-wise linear Galerkin basis functions Φ_*X*,*k*_(*v*), such that $X_{m}\left(v\right)=\sum_{k=1}^{N_{X}}\Phi_{X,k}\left(v\right)\cdot a_{X_{m},k}$ . The degrees of freedom $a_{X_{m},k}$ are extracted with an iterative, greedy algorithm minimizing the L_2_-distance to the data. The number of degrees of freedom *N*_*X*_ is: *N*_*F*_ = 14, *N*_*G*_ = 24, and *N*_*H*_ = 10 in transmural, circumferential and longitudinal direction, respectively. In contrast to a tensor projection that discretizes the data with $N_{F}\cdot N_{G}\cdot N_{H}=3360$ degrees of freedom, the PGD compresses the data by extracting basis functions and therby reduces the number of degrees of freedom to (*N*_*F*_ + *N*_*G*_ + *N*_*H*_) ⋅ *N*_*PGD*_ = 288. In a second step, the variations of these spatial basis functions across hearts are extracted. To this end, first, the mean across all hearts of each resulting 1D function *X*_*m*_(*v*) within the PGD basis was subtracted (*f*_*d*,*m*,*mean*_(*v*) for variable *v* ∈ {*t*, *c*, *l*} and the *m*th PGD basis function of projection *d* ∈ {*t*, *c*, *l*}). Subsequently, a Singular Value Decomposition (SVD) of the 1D functions representing the variations from the mean was applied. The SVD basis functions *f*_*d*,*m*,*n*_(*v*) for variable *v* ∈ {*t*, *c*, *l*}, the *m*th PGD basis function, and *n*th SVD mode were obtained. For each projection *d* = *t*, *c*, *l*, a truncated PGD basis with six modes, represented by a truncated SVD with six modes (Stimm et al., 2022), was included into the model:

$$f_{d;\text{PGD}}\left(t,c,l\right)=\overset{N_{\text{PGD}}=6}{\underset{m=1}{\sum }}[\underset{v=\in \left\{c,t,l\right\}}{\prod }[\left(w_{v,m,mean}\cdot f_{v,m,mean}\left(v\right)+\overset{N_{\text{SVD}}=6}{\underset{n=1}{\sum }}w_{v,m,n}\cdot f_{v,m,n}\left(v\right)\right)]].$$ (4)

The weights *w*_*v*,*m*,*mean*_ and *w*_*v*,*m*,*n*_ were adapted to the first eigenvector of the *in-vivo* cDTI tensor. To this end, a modified Proper Generalized Decomposition was performed directly using the previously derived SVD basis instead of the Galerkin basis.

The POD-model was extracted by applying a Proper Orthogonal Decomposition (Buljak and Maier, 2011; Buoso et al., 2019) directly across the hearts. To this end, the data was mapped onto a common, equidistant grid with 200 circumferential, 20 transmural, and 120 longitudinal points, with discrete coordinates (*t*_*i*_, *c*_*j*_, *l*_*k*_), and: *i* = 1, ., 20; *j* = 1, ., 200; *k* = 1, … , 120. The data was sliced in slices with transmural normal direction and flatted into vectors. All vectors of all transmural positions and all hearts were combined into one matrix. A Proper Orthogonal Decomposition was applied to extract the 2D POD basis functions $\Phi_{m}\left(c_{j},l_{k}\right)$ . The basis was truncated at eight modes (Stimm et al., 2022) and combined with adaptable weights $w_{m,t_{i}}$ , one for each discrete transmural coordinate *t*_*i*_ and POD mode *m*, resulting in the POD-model:

$$f_{d;\text{POD}}\left(t_{i},c_{j},l_{k}\right)=\overset{N_{\text{POD}}}{\underset{m=1}{\sum }}\left[\left(w_{m,t_{i}}\cdot \Phi_{m}\left(c_{j},l_{k}\right)\right)\right].$$ (5)

To adapt the weights to the sparse data, the same common grid was reduced, such that it only contained the closest point to each data point. A tri-linear interpolation of the data onto these remaining grid points was performed. The weights $w_{m,t_{i}}$ were then obtained by applying a gappy POD (Willcox, 2006), only taking the remaining data points into account.

#### 2.2.3 Rule-based method (RBM)

The rule-based method is based on Bayer et al. (2012), but was reduced to the LV only. It is based on rotations of the local coordinate axis defined by the local helix and transverse angle of the fiber direction. Two linear functions of the transmural coordinate (t) define the local helix angle:

$$\alpha \left(t\right)=\alpha_{\mathrm{endo}}\cdot \left(1-t\right)+\alpha_{\mathrm{epi}}\cdot t,$$ (6)

and transverse angle:

$$\beta \left(t\right)=\beta_{\mathrm{endo}}\cdot \left(1-t\right)+\beta_{\mathrm{epi}}\cdot t.$$ (7)

Instead of using fixed parameter values inferred from observations from histology, the parameters *α*_*endo*_, *α*_*epi*_, *β*_*endo*_, and *β*_*epi*_ were adapted to the *in-vivo* cDTI data. To this end, a least squares fit of the two linear transmural functions was performed, one to the helix and one to the transverse angle calculated from the *in-vivo* cDTI.

#### 2.2.4 Fiber and sheet orientation

To generate a microstructure representation with realistic sheetlet orientation, for each of the four fiber models from *in-vivo* cDTI data, the corresponding sheetlet directions were calculated to match a physiological orientation measured by (Nielles-Vallespin et al., 2017), characterized by the E2A angle. The sheet orientation was estimated using the local fiber orientation and physiological coordinate axes, such that a diastolic E2A angle of 13°(Nielles-Vallespin et al., 2017) was obtained. The E2A angle was defined as the angle between the projection of the sheet direction onto the cross-myocyte plane and the cross-myocyte direction (Ferreira et al., 2014). The cross-myocyte direction is the cross-product of the direction of the transmural axis and the projection of the fiber direction onto the local longitudinal-circumferential plane. The cross-myocyte plane is spanned by the cross-myocyte direction and the local transmural axis.

### 2.3 Computational left ventricular model

The computational cardiac biomechanics model was adapted from the Living Heart Human Project (version 2.1, Simulia, Dassault Systèmes) (Baillargeon et al., 2014), previously used for biomechanical simulation studies by (Genet et al., 2014; Sack et al., 2016a,b; Genet et al., 2016; Sack et al., 2018b,a; Peirlinck et al., 2018; Dabiri et al., 2018; Sahli Costabal et al., 2019; Dabiri et al., 2019b,a; Peirlinck et al., 2021; Guan et al., 2020; Sack et al., 2020; Dabiri et al., 2020; Wisneski et al., 2020; Heidari et al., 2022; St. Pierre et al., 2022). The dynamic problem was solved in Abaqus 2020 (Simulia, Dassault Systèmes) using meshes of the LV with 160,000 linear tetrahedral elements with an average edge length of 1.8 ± 0.5 mm. Four different models with varying microstructure orientation (see Section 2.2.4) were set up and the material parameters were adapted (see Section 2.3.4) for each model, respectively.

The behaviour of the myocardium comprises a micro-structurally motivated anisotropic passive response and an active contribution due to active contraction, triggered by electrical activation. The active stress only acts in fiber and sheet direction and is zero in the other directions. The fiber stress ***σ***_**f**_ and sheet stress ***σ***_**s**_ are given by:

$$\begin{array}{cc} \sigma_{f} & =\sigma_{\mathrm{passive,f}}+T_{\mathrm{active}}\vec{f}⊗\vec{f},\\ \sigma_{s} & =\sigma_{\mathrm{passive,s}}+\sigma_{\mathrm{active,s}}=\sigma_{\mathrm{passive,s}}+n\cdot T_{\mathrm{active}}\vec{s}⊗\vec{s}, \end{array}$$ (8)

with active tension *T*_*active*_ (Eq. 12) and unit vectors in fiber $\vec{f}$ and sheet $\vec{s}$ direction. The scaling factor *n* can take values between 0 and one and scales the additional active stress acting in the sheet direction. This additional cross-fiber stress is motivated by the need to model the fiber dispersion (Krishnamurthy et al., 2016). The fiber vector $\vec{f}$ , defined by the local predominant aggregated myocyte orientation, does not take the variation of myocyte orientations within the represented local region into account. The passive Cauchy stress tensor *σ*_*passive*_ is given by:

$$\sigma_{\mathrm{passive}}=2J_{F}^{-1}F\left(\partial \Psi /\partial C\right)F^{T},$$ (9)

with strain energy density **Ψ** (detailed in Section 2.3.1), right Cauchy-Green tensor **C**, deformation gradient **F** and its Jacobian *J*_*F*_. In our model, we assume a synchronous ventricular activation. This simplification is motivated by missing data on the heterogeneously distributed electro-mechanical delays (Kerckhoffs et al., 2003), a Purkinji fiber tree, and tissue conductivity values.

#### 2.3.1 Passive constitutive model

The hyperelastic, nearly-incompressible passive response was represented by an isochoric strain energy ***ψ***_*ic*_ and a volumetric contribution ***ψ***_*vol*_. The isochoric part was described by the orthotropic Holzapfel-Ogden material model (Holzapfel and Ogden, 2009):

$$\begin{array}{ll} \psi_{ic} & =\frac{A\cdot a_{\mathrm{iso}}}{2b_{\mathrm{iso}}}\exp\left[b_{\mathrm{iso}}\left(I_{C_{1}}-3\right)\right]+\underset{i=f,s}{\sum }\frac{A\cdot a_{i}}{2b_{i}}\\ & \;\cdot \left(\exp\left[b_{i}{\left(I_{4i}-1\right)}^{2}\right]-1\right)+\frac{A\cdot a_{fs}}{2b_{fs}}\left(\exp\left[b_{fs}I_{8fs}^{2}\right]-1\right), \end{array}$$ (10)

formulated in terms of the invariants (*I*) of the isochoric, right Cauchy-Green tensor ( $\bar{C}=J_{F}^{-2/3}C$ , with right Cauchy-Green tensor **C** and Jacobian of the deformation gradient *J*_*F*_) and eight material parameters: *a*_*iso*_, *b*_*iso*_, *a*_*f*_, *a*_*s*_, *b*_*f*_, *b*_*s*_, *a*_*fs*_, *b*_*fs*_. The linear parameters *a*_*i*_, with *i* ∈ {*iso* (isotropic), *f* (fiber), *s* (sheet), *fs* (fiber-sheet)} have the unit of stress and the exponential parameters *b*_*i*_ are unitless. Initial values were taken from (Peirlinck et al., 2019): *a*_*iso*_ = 0.0943*kPa*, *b*_*iso*_ = 5.874, *a*_*f*_ = 0.311*kPa*, *a*_*s*_ = 0.0431*kPa*, *b*_*f*_ = 11.271, *b*_*s*_ = 9.772, *a*_*fs*_ = 0.0254*kPa*, *b*_*fs*_ = 2.405. The linear scaling factor *A* was introduced for personalization by Sack et al. (2018a); Peirlinck et al. (2019), as outlined in Section 2.3.4. $I_{C_{1}}=\bar{C}:I$ is the isotropic, isochoric strain invariant, $I_{4f}={\vec{f}}^{T}\bar{C}\vec{f}$ and $I_{4s}={\vec{s}}^{T}\bar{C}\vec{s}$ reflect the transversely isotropic contributions in the fiber and sheet directions and the orthotropic pseudo-invariant $I_{8fs}={\vec{f}}^{T}\bar{C}\vec{s}$ reflects the fiber-sheet interaction. The volumetric part is modeled by:

$$\psi_{\mathrm{vol}}=\frac{1}{D}\left(\frac{\left(J_{F}^{2}-1\right)}{2}-\log\left(J_{F}\right)\right),$$ (11)

with *J*_*F*_ being the Jacobian of the deformation gradient and *D* being the inverse multiple of bulk modulus (with bulk modulus *K* and *D* = 2/*K*), set to *D* = 0.1 as the initial setting of the Living Heart Human Project (version 2.1, Simulia, Dassault Systèmes) (Baillargeon et al., 2014). This small value of *D* = 0.1 was chosen to enforce nearly-incompressible behavior. This approach of prescribing a small value of D to enforce nearly-incompressibility has been previously applied in (Sack et al., 2018a). A small mass proportional Rayleigh damping was used to damp oscillations as in (Sack et al., 2018a). The damping contribution was proportional to the mass matrix of each element with damping factor of 160 [1/s]. The density was set to 1.6 ⋅ 10^3^[*Kg*/*m* ^3^]. These settings were taken from the original Living Heart Human Project model (Baillargeon et al., 2014).

#### 2.3.2 Active model

The active stress (Eq. (12)) follows a time-varying elastance model introduced by Guccione et al. (1995) as implemented in (Walker et al., 2005). The active stress in the fiber direction *σ*_*active,f*_ is a function of the time after activation (t) and the fiber strain (*E*_*ff*_).

$$T_{\mathrm{active}}\left(t,E_{ff}\right)=T_{\max}\frac{Ca_{0}^{2}}{Ca_{0}^{2}+ECa_{50}^{2}\left(E_{ff}\right)}C_{t}\left(t,E_{ff}\right),$$ (12)

$$ECa_{50}\left(E_{ff}\right)=\frac{Ca_{0,\max}}{\sqrt{\exp\left(B\left[l\left(E_{ff}\right)-l_{0}\right]\right)-1}},$$ (13)

$$\begin{array}{cc} C_{t}\left(t,E_{ff}\right) & =\frac{1}{2}\left[1-\cos\left(\omega \left(t,E_{ff}\right)\right)\right]\\ \text{with}\;\omega \left(t,E_{ff}\right) & =\left\{\begin{array}{cc} \pi \cdot \frac{t}{t_{0}}\;\; & \text{if}\;0\leq t<t_{0}\\ \pi \cdot \frac{t-t_{0}+t_{r}}{t_{r}}\;\; & \text{if}\;0\leq t_{0}\leq t<t_{0}+t_{r}\\ 0\;\; & \text{if}\;t_{0}+t_{r}\leq t \end{array}\right. \end{array}$$ (14)

The parameter *T*_max_ is the maximum of the developed tension and acts as a contractility scaling factor. The function *ω*(*t*, *E*_*ff*_) (Eq. 14) differentiates the three phases: increasing active tension after activation, relaxation, and no active contribution after relaxation. Within the time *t*_0_, the active tension follows a cosine-shaped increase. The relaxation time is given by *t*_*r*_ = *ml* + *b*, a linear function of the sarcomere length *l*. The sarcomere length $l\left(E_{ff}\right)=l_{r}\sqrt{2E_{ff}+1}$ changes with fiber strain *E*_*ff*_ and is scaled by the initial sarcomere length *l*_*r*_. Consequently, the function *C*_*t*_ (Eq. (14)) introduces the influence of the sarcomere length on the duration of the contraction. The length-dependent calcium sensitivity *ECa*_50_ is modeled by Eq. (13), with parameter *B* relating the sarcomere length to the peak tension and threshold sarcomere length *l*_0_ below which no force develops. This representation of the calcium sensitivity includes a switch, that is active if $\left(l\left(E_{ff}\right)-l_{0}\right)$ is small, triggering a rapid decrease of the active stress. If the switch is not activated, the active stress decreases with a cosine-shaped relaxation curve. The peak intracellular calcium concentration is given by the parameter *Ca*_0_. The parameter values: *Ca*_0_ = 4.35 *μmol*/*L*, *Ca*_0, max_ = 4.35 *μmol*/*L*, *B* = 4750 *mm* ^−1^ (Walker et al., 2005), *l*_0_ = 0.75 *μm*, and *l*_*r*_ = 1.835 *μm* were taken from the Living Heart Human Project (Baillargeon et al., 2014), the parameters *t*_0_, *m*, *b*, and *T* *_max_* were adapted to the data, as outlined in Section 2.3.4.

#### 2.3.3 Boundary conditions

Motivated by findings of Peirlinck et al. (2019); Asner et al. (2017) that over-constraining the basal plane affects global functional parameters and local strains, the geometric boundary condition applied here allows for circumferential and radial tissue motion at the base. The boundary condition constraining the basal motion is defined in accordance to (Peirlinck et al., 2019) (Case 5). All nodes within the basal plane were constrained in the longitudinal direction and the center of mass of the base was fixed in all directions. A continuum distributed coupling constraint was used to couple the average circumferential and radial displacements of the nodes within the endocardial, basal ring to the fixed center of mass. Consequently, this results in a zero average displacement of the endocardial ring, while all other nodes within the basal plane were only constrained in longitudinal direction.

The hemodynamic loading was represented by a pressure loading condition prescribed at the endocardial surface over the cardiac cycle. A generic left ventricular pressure curve was extracted from the original Living Heart Human Project model (Baillargeon et al., 2014). This pressure curve was then adapted to measured pressure values (Section 2.1.3) while keeping the duration of the diastolic and the systolic part constant (depicted in Figure 1, Box I). This compromise allowed us to keep the parameters of the active material model, that are related to the timing of increase in active tension constant and thereby reduced the complexity of the active fitting problem. To merge the generic and the measured pressure curves, the measured diastolic pressure waveform was scaled in time to the duration of diastole of the generic pressure trace. The systolic pressure waveform of the generic pressure curve was scaled in amplitude such that the peak pressure corresponds to the maximal measured pressure *p*_max_. The transitions between diastole and systole were smoothed, and the waveform was circularly shifted, such that the initial value corresponded to the initial configuration in diastasis. The resulting pressure curve is shown in Supplementary Figure S1.

#### 2.3.4 Estimation of material parameters and unloaded reference state

For each microstructural model, an unloaded reference state as well as passive and active material parameters were personalized using volume and length information of the LV obtained from cine data (Section 2.1.2).

The initial mesh was obtained in diastasis, corresponding to a mid-diastolic time-point (Shmuylovich et al., 2010) and a plateau of the volume curve (Freytag et al., 2021). In early diastole, a steep increase in volume was observed, while after diastasis, the volume increased slowly. This suggests an influence of energy stored in the myocardium in early diastole, compromising the use of the end-systolic configuration and the configuration corresponding to the minimal pressure as an initial configuration. However, the pressure loading in diastasis required the estimation of an unloaded reference configuration. Due to the influence of the material parameters on the unloaded configuration (Nikou et al., 2016a; Hadjicharalambous et al., 2021), a joint estimation is required.

The initial passive parameters were taken from a study with the same material model and basal boundary condition by Peirlinck et al. (2019). One scaling factor *A*, scaling the linear coefficients, was introduced in the passive material model (Eq. 10) and adapted in the fitting process. A second scaling factor *B*, scaling the dimensionless tissue parameters (*b*_*iso*_, *b*_*f*_, *b*_*s*_, *and* *b*_*fs*_), previously used in (Sack et al., 2018a; Peirlinck et al., 2019) was disregarded to increase the time-efficiency of the joint estimation process. This simplification was motivated by findings of Hadjicharalambous et al. (2015) demonstrating a good trade-off between model fidelity and parameter identifiability for a reduced Holzapfel model and fixed values of the dimensionless parameters. The previous adaptation by (Peirlinck et al., 2019) ensures a feasible initial choice, consequently *B* = 1.0 was used for all simulations. This simplification was confirmed by an additional parameter sweep for *A* and *B* using the model with the HFC fiber field. This showed a strong correlation of an increase in *A* with a decrease in *B* and hence a small change in the objective function along a diagonal region was observed in a range of *B* ∈ {0.5, 0.75, 1.0, 1.25, 1.5} and *A* = 16, 17, 18, ., 46. Joint adaptation of *A* and the unloaded reference geometry was performed with a parameter sweep of *A* with a step size of Δ*A* = 1.0. In each iteration, first a suction problem was simulated to obtain an approximation of the unloaded reference. To this end, a negative pressure ramp on the endocardium, with a maximal absolute value of the pressure in diastasis, was applied. Second, a forward simulation to end-diastole was performed. Thereby, a linear pressure increase to diastatic pressure followed by the end-diastolic pressure curve was applied as the loading condition. During this forward simulation, the volumes at the initial diastatic pressure (*V*_*D*,*simulation*_) and at end-diastole (*V*_*ED*,*simulation*_) were obtained and compared to the initial volume in diastasis *V*_*D*_ and the end-diastolic volume *V*_*ED*_ extracted from the cine data. The objective function (*f*_*objective,p*_, Eq. 15) was defined as the sum of the absolute errors:

$$\begin{array}{ll} f_{\mathrm{objective,p}} & =|\left(V_{ED,data}-V_{ED,simulation}\right)|\\ & \;+|\left(V_{D,data}-V_{D,simulation}\right)| \end{array}$$ (15)

The personalized parameter *A* = *A*_*opt*_ and the corresponding unloaded reference configuration were obtained at the minimum of *f*_*objective,p*_. The suction problem, as previously used in the Living Heart Human model (Dassault Systèmes, 2018), was chosen to approximate the inverse mechanics problem within only one simulation, in order to reduce the computational complexity compared to an iterative backward displacement method (Sellier, 2011). A further advantage of this approach is that it can be used without changes to the FEM implementation. The forward simulation with active part included an initial loading with a linear pressure increase from the reference configuration to diastasis and consecutively a cardiac cycle. The initial active material parameters were taken from the Living Heart Human Model (Dassault Systèmes, 2018). The parameters *m* and *b*, that determine the relaxation time *t*_*r*_, and thus influence timing and shape of the relaxation (Eq. 14), were adapted manually to the pressure curve, in order to adapt the point in time when zero activation is reached. This adaptation was required to account for the longer duration of the pressure trace due to the initial loading step and a simplified synchronous electrical activation compared to the Living Heart Human Model (Dassault Systèmes, 2018). The manual fit was performed with the RBM fiber field and the resulting parameters were kept constant for all simulations (*m* = 300, *b* = −0.38). In accordance to previous works, the parameter *T*_max_, scaling the active tension (Peirlinck et al., 2019), and the scaling factor *n*, providing the active sheet contribution (Sack et al., 2018a), were personalized to each model with varying fiber definition. First, the parameter *T*_max_ was adapted using parameter sweeps with manual refinement, such that the end-systolic volume evaluated during early iso-volumetric relaxation at a pressure value of *p* = 60 mmHg coincided with the value extracted from the data (*V*_*ES*_). Next, the parameter *n* was optimized in a parameter sweep between *n* = 0.4 and *n* = 0.9 with a sampling distance of Δ*n* = 0.05. The objective function, defined as the absolute error between the data and the simulation of the maximal left-ventricular shortening during the cardiac cycle with respect to the configuration in diastasis, was minimized. The left-ventricular length was defined as the Euclidean distance between the apex and the center of the base and extracted from each forward simulation. If the minimum was located between two sampling points of the parameter sweep, the optimal parameter *n*_*opt*_ was approximated by linear interpolation. The choice of the objective function was motivated by the work of Sack et al. (2018a) that also included this global metric related to longitudinal strain into the fitting process of the active sheet contribution.

### 2.4 Simulation study

We studied the sensitivity of the simulation output depending on the underlying fiber field obtained from the same *in-vivo* cDTI input, but, computed with different interpolation and mapping methods with varying number of degrees of freedom and varying smoothing strength. To exclude the influence of the material parameters, for each model, simulations with the optimal parameter setting of all other models were conducted and compared. To evaluate the simulation output, the volume over the cardiac cycle was extracted and the pressure-volume relation (pV-loop) was analyzed. Global physiological parameters: end-diastolic volume (EDV), end-systolic volume (ESV), stroke volume (SV), defined as the difference between EDV and ESV, and ejection fraction (EF) were calculated. The circumferential, radial, longitudinal, and fiber strains relative to the initial state in diastasis were extracted. The median over the myocardium during the cardiac cycle and the distribution within the myocardium at peak systole were compared. The twist, defined as the difference in rotation of apex and base, was measured relative to the end-diastolic state.

## 3 Results

**FIGURE 2:**
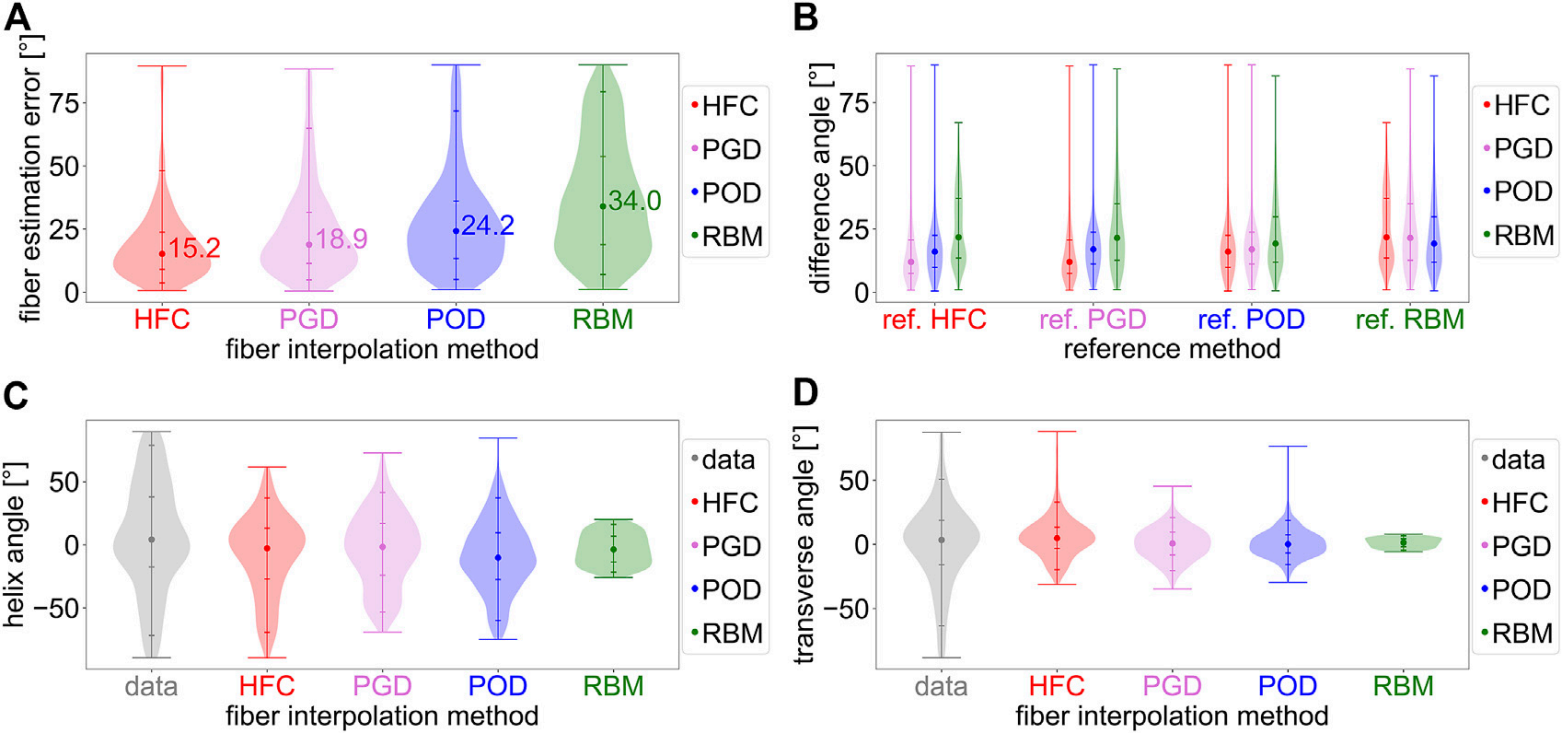
Analysis of the interpolated fiber fields from *in-vivo* cDTI data obtained with the four interpolation methods: the direct tensor interpolation method using heat flux coordinates (HFC) in red, the low rank model estimation based on Proper Generalized Decomposition (PGD) in violet, the low rank model estimation based on Proper Orthogonal Decomposition (POD) in blue, and the rule-based method adapted to the data (RBM) in green. Subplot **(A)** shows the absolute angular error distribution and median value for the *in-vivo* interpolation experiment. The *in-vivo* cDTI data is interpolated to a previously excluded mid-ventricular short-axis slice and compared to the measured cDTI data. Subplot **(B)** analyses the distributions of the mutual angular difference between the resulting interpolated fiber fields. The reference fiber field is noted as label on the horizontal axis. Subplots **(C,D)** depict the distributions of the helix and transverse angle obtained from the data (gray) and the interpolated fiber fields.

In Figure 2, the interpolation errors were compared for the different *in-vivo* fiber models based on the leave-one-out technique with a mid-ventricular short-axis slice of the cDTI data as target (Section 2.2). Figure 2A shows the distributions of the interpolation error. The median interpolation error is the smallest for the HFC method (15.2°), followed by the PDG (18.9°), and POD models (24.2°). The highest error is observed for the RBM (34.0°). To assess the similarity of the fiber fields, the distributions of the mutual differences are shown in Figure 2B. The fiber field obtained from HFC is most similar to the fibers obtained from PGD and *vice versa*. Both show smaller median angular differences to the fiber field resulting from POD than compared to RBM. Remarkably the difference in angle distribution when comparing HFC to RBM is skewed with a peak at the 25th percentile and a tail of higher difference angles leading to a smaller overall spread. All other distributions have outliers at higher difference values. When comparing the fiber field of the POD model to the fibers from HFC and PGD, the distributions are similar. A slightly higher difference is observed when compared to RBM with higher median and 75th percentile. When comparing all fiber fields to RBM, the POD method shows the largest degree of similarity with the smallest median and 75th percentile.

Figures 2C,D show the histograms of the characteristic helix and transverse angles from the four interpolated fiber fields compared to the *in-vivo* data. The fibers obtained from the HFC interpolation show the most similar distribution compared to the data for negative helix angles. The positive helix angles, corresponding to the subendocardial layer, are underestimated by 41.7°/52.8% for the 95-percentile. The helix angle distributions of the fiber fields, resulting from the PGD, POD, and RBM models, underestimate high positive (for the 95th percentile by PGD: 47.3%/POD: 52.9%/RBM: 79.3%) and negative (for the fifth percentile by PGD: 25.8%/POD: 16.5%/RBM: 69.9%) helix angles. The fiber field from RBM has the smallest spread in helix angle. The fibers from the POD model have a bias towards negative helix angles with a negative median of −10.2°. For all other interpolated fiber fields, the negative bias is smaller, with medians between −1.6° and −3.6°. The data has a small imbalance towards positive values with a median of 4.3°. As depicted in Figure 2D, the spread of the transverse angle distribution is smaller for all interpolated fiber fields compared to the data, with the histogram of the RBM model showing the smallest spread. The shape of the distribution is most similar to the data for the HFC-model and PGD-model, and more similar for the POD-model than the RBM-model.

**FIGURE 3:**
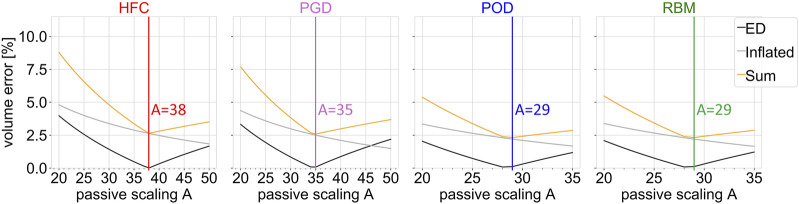
Objective function (sum; yellow) of the joint estimation of the passive material parameter and unloaded configuration, together with the single volume error contributions as function of the passive scaling parameter A. The ventricular volume of the simulation results is compared to the volume obtained from cine MRI data at the configuration inflated to the initial pressure (inflated; gray) and at the end-diastolic configuration (ED; black). Each subplot shows the result of the parameter sweep for one interpolated fiber field obtained by the four compared methods: HFC, PGD, POD, and RBM. The vertical lines indicate the optimum.

Figure 3 shows the objective function (yellow line) of the joint estimation of the passive scaling parameter *A* and the unloaded reference configuration (outlined in Section 2.3.4), evaluated in a parameter sweep of *A*, between *A*_*lb*_ = 20 and a variable upper boundary between 35 and 50, adapted to the stiffness of the model. The two contributions to the objective function are shown: the volume error at the initial diastatic pressure during inflation (inflated/gray line) and the volume error in end-diastole (ED/black line). The volume error, corresponding to geometry at the initial diastatic pressure during inflation, decreases for all models with increasing stiffness (increasing *A*). The volume error for end-diastole shows a minimum in the range of investigated values for *A*. The resulting optimal passive stiffness scaling factors *A* are listed in Table 1.

**TABLE 1:** Personalized passive and active tissue parameters (A, *T*_max_) and the active contribution in sheet direction (n) for the four models with different fiber orientation obtained by the four interpolation methods: HFC-model, PGD-model, POD-model, and RBM-model.

optimized parameter	HFC-model	PGD-model	POD-model	RBM-model
passive scaling factor (*A*)	38	35	29	29
active tension scaling factor (*T*_max_)[*MPa*]	0.105	0.109	0.08	0.071
active contribution in sheet direction (n)	0.68	0.7	0.76	0.7

The optimized scaling factor of the active tension *T*_max_ is listed in Table 1. The mutual differences show the highest similarity in personalized *T*_max_ between HFC-model and PGD-model, followed by POD-model, and RBM-model.

**FIGURE 4:**
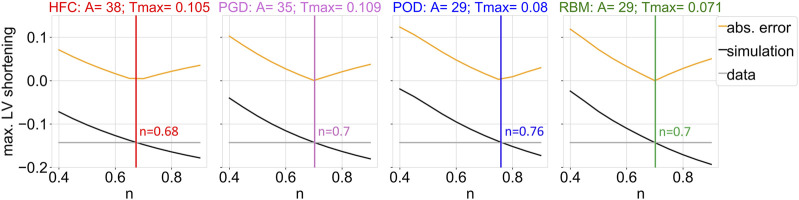
Objective function (yellow) of the fitting procedure of the parameter that scales the active contribution in sheet direction (n), which is given by the difference between data and simulation outcome of the maximum of the left ventricular shortening during the cardiac cycle with respect to the initial configuration in diastasis. The maximum of the left ventricular shortening as a function of the parameter n is shown, for the data (gray) and the simulation output (black), together with their absolute difference (yellow). The optimized parameter value resulting in the smallest absolute shortening error is indicated by the vertical line. Each subplot shows the results of the parameter estimation for one interpolated fiber field obtained by the four compared methods: HFC, PGD, POD, and RBM. The subtitles indicate the previously adjusted parameter values of the passive (A) and active (*T* *_max_* [*MPa*]) scaling factors.

Figure 4 depicts the personalization of the active tension in sheet direction (*n*). The gray line depicts the maximal LV shortening obtained from the CINE data. The black line shows the maximal LV shortening in the simulation as a function of the parameter n. The yellow line corresponds to the objective function, defined as the absolute difference of the maximal long axis shortening between simulation and data. The simulated maximal LV shortening decreases monotonically with increasing n. Small values of active sheet contribution, underestimate absolute shortening, while high values lead to an overestimation of absolute shortening. The optimal values of n are listed in Table 1.

**FIGURE 5:**
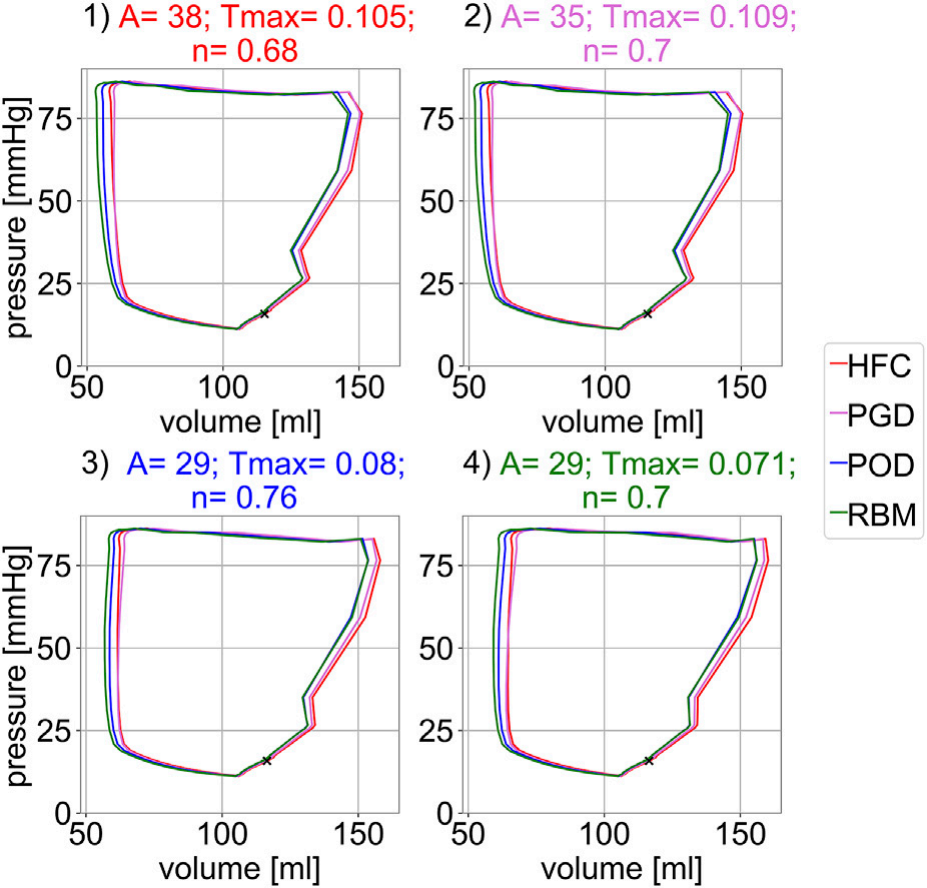
Simulated pressure-volume relation for the four different parameter settings shown in the four subplots. Each parameter settings was optimized to one interpolated fiber field obtained with the four compared methods: 1) HFC, 2) PGD, 3) POD, 4) RBM. For each parameter configuration (subplot) the pV-loop for all four interpolated fiber fields are depicted. The marker indicates the diastatic state, i.e. the initial state of the simulation.

Figure 5 shows the pressure-volume relations of the forward simulation starting in diastasis, after prior inflation from the unloaded reference configuration. The four subplots correspond to the four parameter configurations obtained from personalization to the four fiber models. For each parameter setting, simulations with all four models, were performed. No sharp isovolumetric contraction is present but rather an indentation of the pv-loop is found. This non-physiological behaviour is related to an imbalance of the increase in pressure and active tension and observed for all models and parameter configurations. The decrease in volume directly after the end-diastolic state is less pronounced for Settings three and four obtained from personalization to the POD-model and RBM-model. In each subplot, the pressure-volume loops are similar for all four models. Their mutual similarity is highest between the HFC-model and the PGD-model and between the POD-model and the RBM-model. The end-diastolic volume (EDV) shows only small differences (below 3 ml) between the models within each setting. The highest EDV is observed for the HFC-model, followed by PGD-model, POD-model, and RBM-model. The EDV for POD-model, and RBM-model are similar with differences <0.2 ml. The order corresponds to the decrease of the personalized stiffness scaling parameter *A*. For all parameter configurations, the smallest end-systolic volume at the beginning of isovolumetric relaxation is observed for the RBM-model, followed by slightly higher values for POD, HFC and PGD model, with differences ≤7 ml or < 13%. The order by increasing end-systolic volume corresponds to the order by increasing active tension scaling factor *T*_max_, obtained from personalization. The same order applies to the stroke volume and ejection fraction (2). The global physiological parameters, minimal ESV, EDV, SV and EF are listed in Table 2. The maximal variation between the models for each parameter settings is < 6 ml (equivalent 8%) for SV and ≤5 percentage points (equivalent 9%) for EF. A high similarity of the global physiological parameters, resulting from the models simulated with their individually optimized parameter setting, is observed with maximal variation of 1.3 *ml* (equivalent 1.8%) in SV and 0.9 percentage points (equivalent 1.6%) in EF.

**TABLE 2:** Simulated global physiological parameters: minimal end-systolic volume (ESV), end-diastolic volume (EDV), stroke volume (SV), and ejection fraction (EF). Each was evaluated for all four models: HCF, PGD, POD and RBM obtained from simulations with all four parameter configurations.

	HFC-model	PGD-model	POD-model	RBM-model
parameter setting HFC: A = 38, *T*_max_ = 0.105 MPa, *n* = 0.68				
EDV [ml]	131.8	130.8	129.2	129.2
ESV [ml]	58.2	59.8	55.5	53.2
SV [ml]	73.5	70.9	73.7	76.0
EF [%]	55.8	54.2	57.0	58.9
parameter setting PGD: A = 35, *T*_max_ = 0.109 MPa, *n* = 0.7				
EDV [ml]	132.5	131.5	129.8	129.9
ESV [ml]	56.7	58.4	54.1	51.9
SV [ml]	75.7	73.1	75.7	78.0
EF [%]	57.2	55.6	58.3	60.1
parameter setting POD: A = 29, *T*_max_ = 0.08 MPa, *n* = 0.76				
EDV [ml]	134.2	133.1	131.4	131.5
ESV [ml]	61.6	61.7	58.7	56.8
SV [ml]	72.5	71.4	72.8	74.6
EF [%]	54.1	53.6	55.4	56.8
parameter setting RBM: A = 29, *T*_max_ = 0.071 MPa, *n* = 0.7				
EDV [ml]	134.2	133.1	131.4	131.5
ESV [ml]	64.6	64.0	61.2	59.3
SV [ml]	69.6	69.1	70.2	72.2
EF [%]	51.9	51.9	53.5	54.9

**FIGURE 6:**
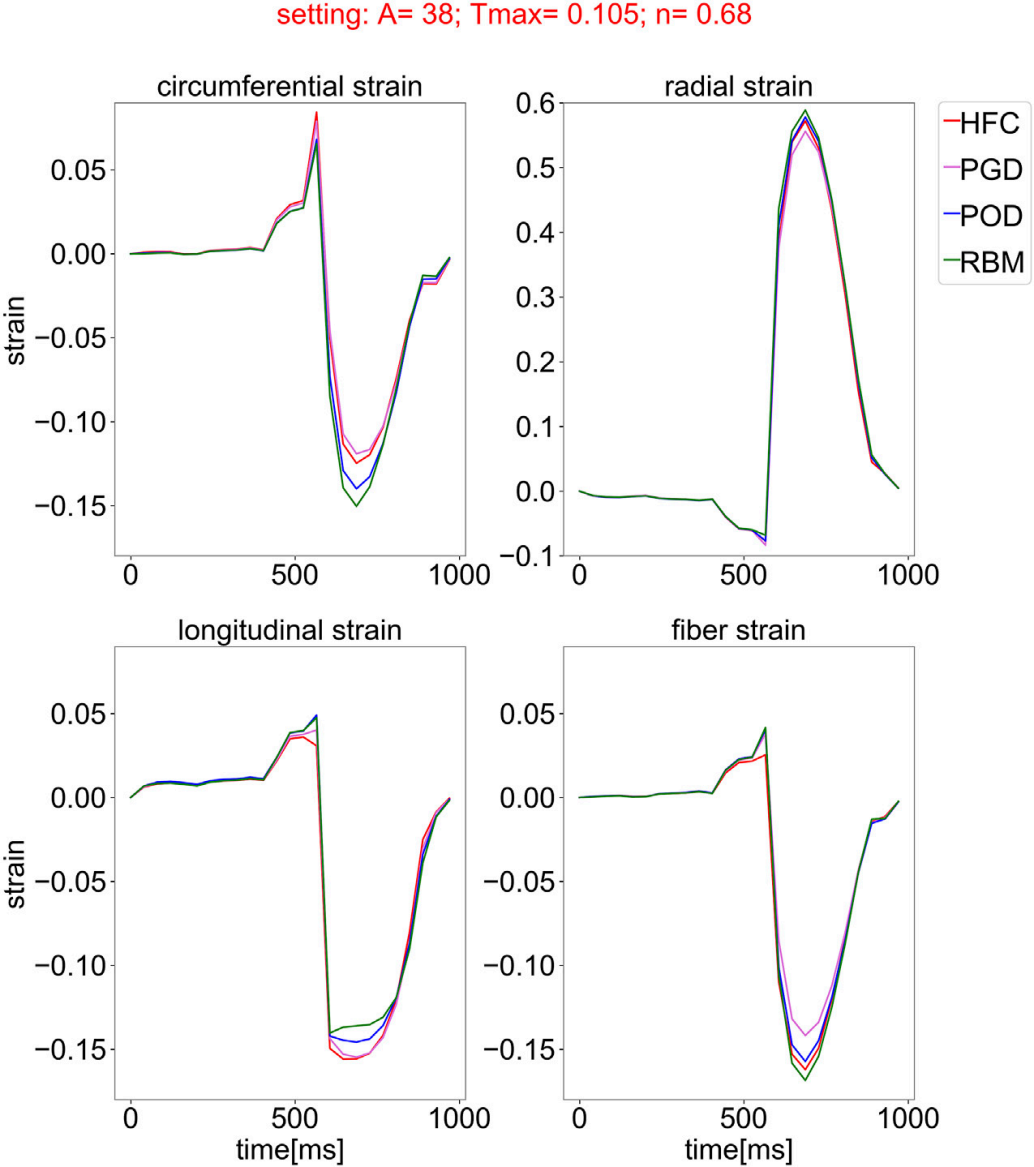
Simulated strain curves over the cardiac cycle, starting from the initial state in diastasis for all models with the four different fiber fields, obtained by the compared interpolation methods (HFC: red, PGD: violet, POD: blue, and RBM: green) and simulated with one exemplary parameter setting optimized for the fiber field interpolated with the HFC method. The subplots show the median of the circumferential, radial, longitudinal, and fiber strain over the myocardium, relative to the inflated state in diastasis.

Figure 6 shows an example time course of circumferential, radial, longitudinal, and fiber strain during the cardiac cycle, starting in diastasis. The strain values are relative to the diastatic state. The presented traces of all fiber models are exemplary for the parameter setting personalized to the HFC-model and are similar in shape for all other parameter settings. During diastole, all strains show only small changes within the first 400 ms after diastasis, corresponding to a plateau in the pressure curve. In late diastole, at the time point t = 400 ms the pressure starts to increase and reaches the end-diastolic pressure (EDP) at t = 514 ms. In parallel the circumferential, longitudinal, and fiber strain increase and the radial strain decreases. The end-diastolic median strains averaged over the models are: circumferential: 0.029, radial: 0.060, longitudinal: 0.038, fiber: 0.023, with a maximal variation between the models smaller than 0.005 for all strains. After end-diastole, during the steep pressure increase, a spike in all strain curves is observed and is most pronounced for the circumferential strain. Similar to the indentation of the pV-loop during iso-volumetric contraction, this behaviour is attributed to the imbalance of active tension and pressure increase (see Figure 5). This spike is followed by rapid decrease in circumferential, longitudinal, and fiber strain, and rapid increase in radial strain, corresponding to systolic contraction. During relaxation and subsequent early diastolic pressure increase, the circumferential, longitudinal, and fiber strain decrease and radial strain increases to the initial configuration with zero strain relative to diastasis.

**FIGURE 7:**
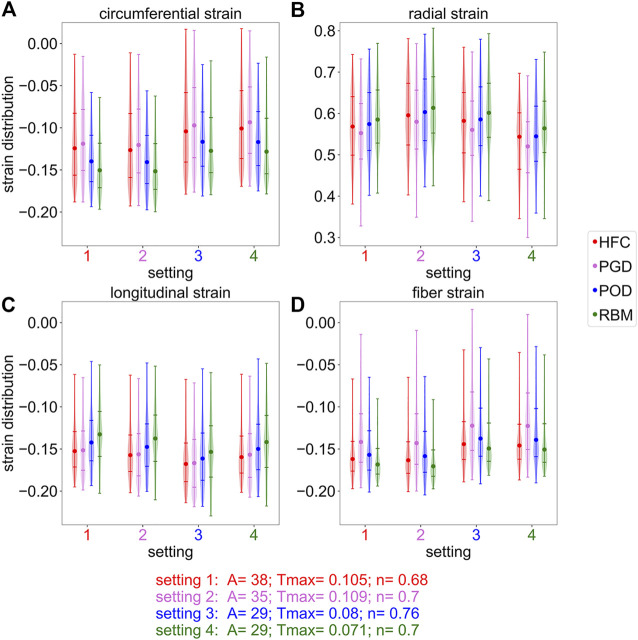
Distribution of the simulated strains in systole, cropped at the 5th and 95th percentiles. The subplots correspond to the circumferential **(A)**, radial **(B)**, longitudinal **(C)**, and fiber **(D)** strain at the end-systolic time-point assigned to the peak radial strain. Each subplot shows four strain distributions (one for each interpolated fiber field) for four different parameter settings (labeled on the *x*-axis), respectively. The parameter settings are obtained by optimization with one interpolated fiber field: 1) HFC, 2) PGD, 3) POG, 4) RBM. Round markers indicate the median, horizontal lines indicate the fifth,25th,75th, and 95th percentile.

Figure 7 shows the peak systolic strain distributions, clipped at the 5th and 95th percentile, for the circumferential, radial, longitudinal, and fiber strains relative to the initial diastatic state in the four subplots. Each subplot depicts the simulation results obtained with all four personalized settings, indicated by the numbers on the horizontal axis. For each setting, the distributions of the simulated strains are shown for all models with the different fiber fields (HFC-model in red, PGD-model in violet, POD-model in blue, RBM-model in green). When comparing the results between the models, the same trends exist for all settings, however, an offset of the strain values between the settings is observed. Also the same variation between the models in the spread of the distributions is observed for all settings.

For the circumferential strain, shown in Figure 7A, the spread of the distribution is wider for the HFC-model and the PGD-model than for the POD-model and the RBM-model. The absolute median strain value is the smallest for the PGD-model, followed by smaller absolute strains for the HFC-model, the POD-model, and RBM-model. This order coincides with the order of decreasing value of *T*_max_ obtained during personalization. The same order, with lowest absolute strain for the PGD-model and highest for the RBM-model, is present for the radial strains as shown in Subplot (B). The median strain values are more similar, when comparing the models with their individually personalized setting (HFC-model/PGD-model/POD-model/RBM-model: circumferential: 0.12/-0.12/-0.12/-0.13; radial: 0.57/0.58/0.59/0.56; Supplementary Figure S2). For the longitudinal strain, shown in Subplot (C), the absolute median value of the HFC-model is slightly higher than for the PGD-model. Lower absolute values are obtained with POD-model, and RBM-model. For the personalized settings for each model, the RBM-model also results in the smallest absolute median value (HFC-model/PGD-model/POD-model/RBM-model: longitudinal: 0.15/−0.16/−0.16/−0.14). For the median absolute fiber strain, shown in Subplot (D), the highest value is observed for the RBM-model, followed by the HFC-model, the POD-model, and the PGD-model. The spread between the 25th and 75th percentile is the smallest for the RBM-model, followed by a slightly higher spread for the HFC-model, higher spread for the POD-model, and largest spread for the PGD-model. The fiber strain with the personalized setting, respectively is: HFC-model/PGD-model/POD-model/RBM-model: 0.16/−0.14/−0.14/−0.15.

**FIGURE 8:**
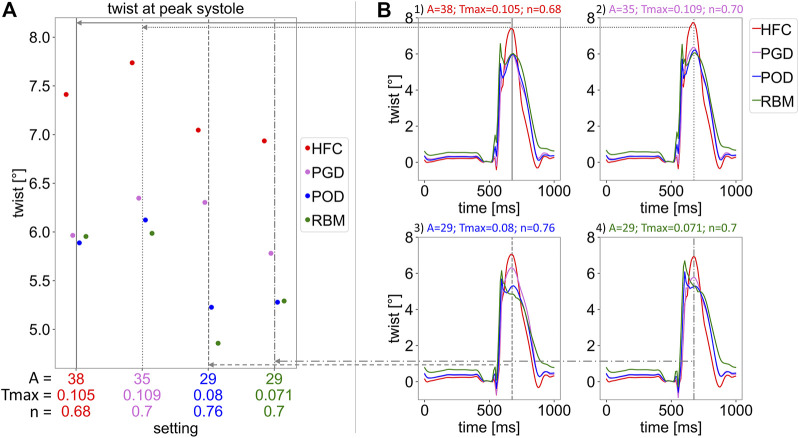
Twist relative to end-diastole. Subplot **(A)** shows the twist in end-systole at 675 ms after the initial state in diastasis, for the four parameter settings listed on the horizontal axis and marked by vertical, gray lines. Each setting was obtained by parameter optimisation with one interpolated fiber field (with interpolation methods: HFC (red), PGD (violet), POD (blue), and RBM (green)). For each setting, simulations with each fiber field, obtained by the four interpolation methods, were performed. The resulting twist is indicated by the 4 markers for each parameter setting. In Subplot **(B)**, the twist over the cardiac cycle is shown. Each Subplot (1–4) corresponds to one parameter setting (optimized to: 1: HFC, 2: PGD, 3: POG, 4: RBM), respectively. All plots show the twist for each interpolated fiber field. The gray vertical lines indicate the time point evaluated in Subplot **(A)**.

In Figure 8, the twist relative to end-diastole is shown. Figure 8B depicts the twist over the cardiac cycle starting from the initial diastatic time point. Figure 8A shows the corresponding peak twist in systole corresponding to the time point of 675 ms after diastasis. In Figure 8B, the four subplots correspond to the four parameter settings obtained by personalization to the four models. The vertical lines indicate end-systole and correspond to the settings illustrated in Subplot (A). For all parameter settings the HFC-model results in the highest end-systolic twist (at t = 675 ms), followed by the twist of the PGD-model. The POD-model and RBM-model result in similar twist as the PGD-model for Setting one and lower twist for the other settings at t = 675 ms. When comparing the twist of the models simulated with their personalized settings, the trend remains (twist: HFC-model: 7.4°; PGD-model: 6.4°; POD-model: 5.3°; RBM-model: 5.3°; Supplementary Figure 2S). A variation of the shape of the twist curve over the cardiac cycle and the value of the twist in end-systole is observed between the settings. When analyzing the change of twist over time in Subplot (B), for all settings, first untwist is observed between diastasis and end-diastole. The decrease in twist in end-diastole (the reference frame for twist calculation) occurs parallel to the EDP increase. This is followed by an oscillation at t = 535 ms. Subsequently, 575 ms after diastasis, during the pressure plateau and ejection, a steep increase in twist is observed with clockwise rotation of the base and counter-clockwise rotation of the apex. At the peak of the clockwise rotation of the base, a distinct spike in twist is present for the POD-model and RBM-model and less pronounced for the PGD-model and HFC-model at the t = 625 ms. Subsequently, twist further increases for the PGD-model and HFC-model, until the maximal apical counter-clockwise rotation is reached at the time point 675 ms after diastasis, corresponding to the end of the pressure plateau. This time point is marked by a vertical gray line and evaluated in Subplot (A). For the POD-model and RBM-model, this further end-systolic twist is not observed for Setting four and also not observed for the RBM-model with Setting 3.

## 4 Discussion

We provide a workflow to equip a biomechanical left-ventricular model with individual microstructure from *in-vivo* cDTI data and have demonstrated for the first time, that integration of individual fiber orientation from sparse *in-vivo* cDTI data in a personalized model is feasible. Four methods with varying fidelity, different amount of smoothing strength, and representation error were applied to bridge the gap between sparse *in-vivo* data and the full field required for computational simulations. The sensitivity of simulation outputs to the interpolation method, was quantified. To this end, the physiological parameters (EDV, ESV, SV, and EF), global strains, systolic strain distribution, and ventricular twist were compared. The models with different data-based fiber fields were personalized based on MRI data, the effect on the personalized parameters of the model was shown and remaining differences in simulation outputs were evaluated.

For all four parameter settings, the differences in simulation results between the four fiber models follow the same systematic trend. This indicates that these variations in simulation results arise from the fiber representation (maximal difference were: EDV: 2.1%; ESV: 12.5%; SV: 7.2%; EF: 8.7%; median strains: circumferential: 37.4%; radial: 8.4%; longitudinal: 15.0%; fiber: 22.9%). The trend between the fiber models and the pair-wise similarity (between HFC-model/PGD-model and between POD-model/RBM-model) is correlated with the error introduced by the interpolation model. This suggests, that the error in the fiber representation is propagated to the simulation output and introduces a systematic bias.

The personalization of the material parameters, scaling the stiffness of the myocardium (A), maximal active tension (*T*_max_), and dispersion (n), showed a dependency on the choice of fiber interpolation method. The parameter values differ significantly between the microstructural models with a maximal increase of 31% for A, 53.5% for *T*_max_, and 11.8% for n. This dependence of stiffness and stress on the fiber orientation has been previously observed by Wang et al. (2013). The variation in parameter values for different interpolation methods hinders comparability of model parameters to physical quantities measured in laboratory experiments (e.g. stiffness or active tension) and moreover requires consideration when comparing modelling studies (e.g. active fiber stress). The parameters not only reflect the physical tissue property but also act as auxiliary parameters to compensate for inaccuracies in the underlying fiber representation. The similarity of the parameters A and *T*_max_ between the HFC-model and the PGD-model is correlated to the highest similarity in the interpolated fiber field providing the smallest interpolation errors compared to the data. The POD-model and RBM-model, both result in higher difference angles compared to the data and lower parameter values. This trend and separation into two groups is the same as observed in the simulation outputs when keeping the parameter setting constant. ESV, median circumferential, and radial strain follow the trend of the resulting personalized *T*_max_. EDV follows the resulting personalized stiffness scaling A.

The analysis for all parameter settings reveals a systematic bias due to the underlying fiber interpolation method. However, in practice the parameters are optimized to the fiber field. This on the one hand results in differences in the personalized parameters and on the other hand in remaining effects on the simulation output. After personalization of each model individually, the differences in median strain values were reduced to: circumferential: ≤ 10.1%; radial: ≤ 3.9%; longitudinal: ≤ 13.8%; fiber: ≤ 17.7%. Differences in the distribution of the fiber strain were observed. A smaller spread of the distribution is present for the HFC-model and the RBM-model compared to the PGD-model and POD-model, resulting from the smoother microstructure representation. Due to the personalization with objective functions including the EDV and ESV (evaluated during relaxation at a pressure of 60 mmHg), the remaining variation in SV and EF are small (SV: 1.3*ml*/1.8%; EF: 0.9 percentage points/1.6%) and can be attributed to minor volume changes during relaxation (Supplementary Figure S2). Compared to literature values all models resulted in circumferential, longitudinal, and fiber strains within a physiological range. (The average median strains were: circumferential: 0.1475, radial: 0.575, longitudinal: 0.153, fiber: 0.148.) Literature values of global strains from porcine studies are: circumferential: 0.17/−0.14/−0.14/−0.15; longitudinal: 0.1675/−0.17/−0.11/−0.13; radial: 0.435/0.65/0.21/0.39 from: (Stoeck et al., 2021): (cine SSFP, mean value)/(Berberoğlu et al., 2022) (cine SSFP)/(Ferreira et al., 2018) (DENSE MRI, mean value)/(Verzhbinsky et al., 2020) (DENSE MRI, median value)), and fiber strain: 0.14 from (Verzhbinsky et al., 2020) (combined DENSE and cDTI). It is noted that a small underestimation compared to the literature values is expected due the difference in the reference configuration: The strains were referred to mid-diastole for this simulation study and end-diastole for the data. The simulated radial strain is higher than three out of four literature values, thus, it might be overestimated in the simulation. However, the literature values of radial strain are subject to a high variation, corresponding to high uncertainty in the data. Furthermore, different approaches for strain estimations, e.g., 2D vs. 3D, are found in literature.

All models underestimated twist (HFC-model/PGD-model/POD-model/RBM-model: twist = 7.4°/6.4°/5.3°/5.3°, corresponding to a maximal torsion of 0.15°/mm, obtained with the HFC-model), compared to literature values measured by 3D tagging with CSPAMM (Rutz et al., 2008) in pigs: twist: 11.05°(Berberoğlu et al., 2022); torsion: 0.27°/mm (Stoeck et al., 2021) averaged over all measurements and all healthy animals. In contrast to twist, torsion excludes the bias of heart size (Young and Cowan, 2012) and is calculated by the ratio of the twist and the end-diastolic ventricular length. The HFC-model led to the highest end-systolic twist, closest to physiological values, followed by the PGD-model and lower values for the POD-model and the RBM-model. This trend agrees well with the interpolation error of the models and unlike other physiological readouts is not compensated by personalization based on volume and LV length data. Similarly, higher twist with a more realistic representations of microstructure has been observed by Gil et al. (2019). Comparing the temporal course of twist during the cardiac cycle to a healthy physiological curve shape (Rüssel et al., 2009; Omar et al., 2015), the twist obtained with the HFC-model and the PGD-model have a more physiological shape, showing less pronounced non-physiological oscillations and spikes during isovolumetric contraction and early systole than the POD-model and the RBM-model. For both, HFC-model and the PGD-model twist increased during ejection after the maximum counter-clockwise rotation of the base and a peak in twist at end-systole is observed as found in physiology. Contrary, the POD-model and RBM-model showed a drop in twist after the early-diastolic spike.

The tensor interpolation method (HFC) corresponds to the smallest interpolation error. It exploits the high spatial coherence of the tensor field in shape adapted coordinates. For optimal performance this method requires the adaptation of the weighting matrix *H* for the anisotropic Gaussian kernel according to the spatial resolution and coverage of the input data. The two low-rank models (PGD and POD) are based on basis functions that were extracted from high-resolution data. This prior information results in a lower degree of flexibility to represent the input data compared to the direct tensor interpolation. The PGD model uses independent basis function in each spatial direction and thus better adapts to the input data compared to the POD model. This results in a smaller interpolation error with the PGD model than with the POD model. The rule-based method is a simple linear model with only four degrees of freedom. Thus, it over-smooths the fiber field and results in the highest interpolation error. The computational cost to generate the personalized fiber fields was: HFC: 21s, PGD: 34s, POD: 18s, RBM: 2s. Times were measured on an eight-core Intel Core i7-10700K, 3.79 GHz desktop computer. The times are three orders of magnitude smaller than the simulation time: HFC: 4 h 39 min, PGD: 4 h 42 min, POD: 4 h 32 min, RBM: 4 h 38 min. The standard deviation of 4.2 min between the simulations for the four models is negligible compared to the total simulation time and no differences in stability were present.

This study has shown the feasibility of personalization of microstructure in a biomechanical model based on a pre-clinical animal experiment with cDTI data including nine short-axis slices. In a more clinical setting, three slices are currently acquired (Khalique et al., 2020, 2018; Gotschy et al., 2021). While full coverage and isotropic spatial resolution are desirable for biomechanical modelling of the heart, cDTI in clinical practice suffers from long scan duration (Nguyen et al., 2020), which is being addressed as part of ongoing imaging research (Nguyen et al., 2021). Due to a steep, non-linear error increase for less than five short-axis slices for all interpolation methods Stimm et al. (2022) (average error increase from nine to three slices in an *ex-vivo* experiment: HFC: 4.6°/PGD: 10.6°/POD: 5.2°/RBM: 0.2°), further development in clinical data acquisition is required. When applying the interpolation approaches presented in this study to clinical data, an additional uncertainty in the simulation results would be present due to this error increase for less than five short-axis slices. However, the tensor interpolation method (HFC) remains the interpolation method with the smallest interpolation error also with three input slices (with an advantage of a 6.7°smaller error compared to the rule-based method (RBM) on average (Stimm et al., 2022)). In this study, we showed that a systematic bias in the simulation results was introduced by the interpolation method and that a correlation of the trend with the increase in interpolation error exists. Combining these two observations of 1) the smaller interpolation error that was observed for the HFC method and 2) the systematic bias of the simulation outputs, we expect a reduction in the bias of the simulation results when using the HFC method also with clinical data. However, the overall higher interpolation error increases the uncertainty compared to the pre-clinical setting and the difference in interpolation performance between the methods deceases in the clinical setting thus the bias is expected to be smaller. To reduce the uncertainty for patient-specific simulations based on clinical *in-vivo* cDTI data, advancements in clinical DTI that would enable a minimum of five input slices are required.

*In-vivo* cDTI studies have observed a reorientation of microstructure in pathology, such as dilated cardiomyophathy (DCM) (von Deuster et al., 2016a), hypertrophic cardiomyopathy (HCM) (Ferreira et al., 2014; Das et al., 2022) and aortic stenosis (AS) (Gotschy et al., 2021). Patient-specific modelling based on a patient-specific microstructure enables to perform modelling studies investigating the link between structure and function of the heart. This can improve the understanding of the mechanical conditions that lead to structural remodelling, and consequently disease progression. Remodelling might onset in an early disease state (Gotschy et al., 2021) and therefore early biomarkers can be revealed by investigation of remodelling. Together with future advances in both *in-vivo* cDTI and patient-specific modelling based on clinical data predictive modelling is a future goal.

Systematic trends of the simulation results were observed between the fiber models, when using the same model parameters. The correlation of this systematic bias with the interpolation performance suggests that a more realistic representation of *in-vivo* microstructure affects the simulation output and therefore reduces the uncertainty in the simulation results. This suggests that using the interpolation method with the lowest interpolation method (HFC tensor interpolation) leads to the simulation results with the lowest uncertainty. The higher twist observed with the HFC-model, remaining present after personalization, further supports that models profit from more realistic representation of microstructure.

## 5 Limitations

In this study, we have investigated the dependence of model behaviour on interpolation errors and compared the results to cohort values. The study concentrated on the isolated bias introduced by the representation method to personalize the model using *in-vivo* cDTI data. The direct comparison to patient-specific physiological measurements is currently not feasible. Results would be biased by model inaccuracy, due to simplifications, such as neglecting the influence of the right ventricle and data uncertainty (e.g. for radial strain and consequently fiber strain estimation) on the individual basis. Further, the improvement compared to a generic fiber representation was not evaluated, which would require a cohort study with enough cases to represent the fiber variability across the population. In pathology, microstructural remodelling is observed (Ferreira et al., 2014; von Deuster et al., 2016a; Gotschy et al., 2021), consequently the variability of structure increases, rendering patient-specific microstructure more important.

The personalized microstructure, based on *in-vivo* cDTI data, is subject-specific, but both, the data acquisition and the interpolation techniques introduce errors. The error of the first eigenvector of the *in-vivo* diffusion tensor, induced by the measurement, can be estimated with the cone of uncertainty (Aliotta et al., 2018). Aliotta et al. (2018) estimated an uncertainty of 15.5°(human data). We estimated an uncertainty of 11.3°in our previous work (Stimm et al., 2022) for porcine data, which was equivalently used in this study. The interpolation error calculated on a mid-ventricular slice was: HFC: 15.2°, PDG: 18.9°, POD: 24.2°, RBM: 34.0°. Sensitivity studies by Geerts et al. (2003); Pluijmert et al. (2017) have found that a difference angle of 8°already influences the simulation output. Consequently, the error minimization by using a HFC representation compared to a RBM representation has an effect on the simulation results, as was confirmed in this study. The analyzed interpolation error reflects the difference angle to the measurement, that is subject to noise and imaging artifacts. Consequently, denoising by smoothing or by exploiting prior information included in the fiber models, also contributes to the measured angular difference. The interpolation performance of the models has been evaluated in detail in (Stimm et al., 2022), presenting an average interpolation error of: HFC: 13.7° ± 1.1°, PGD: 17.8° ± 5.2°, POD: 19.0° ± 3.3°, RBM: 24.8° ± 6.0°*in-vivo*. Thus, the interpolation error of the underlying case is slightly above the average error for all methods. The number of degrees-of-freedom of the data-based models (PGD and POD) was adapted to *ex-vivo* data Stimm et al. (2021). Optimization in an *in-vivo* study might increase denoising and result in smoother microstructure representation, thereby, potentially reducing the spread of the strain distributions. All methods were adapted based on all data points, however, the endo- and epicardial boundaries were subject to partial-volume effects, resulting in higher uncertainty of these data points. Further, segmentation errors, in the proximity of the papillary muscles at the endocardium, might have influenced the errors at the boundaries. This resulted in wider spread of the helix and transverse angles in the data. This spread is reduced by denoising effects of the interpolation. Although, over-smoothing has the same effect, it reduces the spread of the helix and transverse angle distributions of the interpolated fiber representations too radically. Adaptation of the fitting such that it only uses data in areas not prone to partial voluming and segmentation errors, might reduce the representation error, especially for the RBM-method with only four degrees of freedom. While the HFC-model resulted in a globally more heterogeneous microstructure than the RBM-model, both result in smoother fiber representations than the PGD-model and POD-model. This might be a reason for the more narrow distribution of the fiber strain found in this study.

The patient-specific model used in this work contains simplifications, influencing the accuracy of the simulations. While right-ventricular deformation and pressure influence left-ventricular mechanics (Palit et al., 2015), a LV only model was used in this study because *in-vivo* microstructure is currently not available for the thinner right-ventricular wall. Further, the surrounding of the heart was modeled by a boundary condition constraining the base as proposed by Peirlinck et al. (2019). Adding a ribcage and pericardial boundary condition can improve model accuracy (Pfaller et al., 2019; Ponnaluri et al., 2019; Strocchi et al., 2020; Hadjicharalambous et al., 2021). However, both, neglecting influences of the RV and the surrounding are expected to result in a consistent offset of the simulation results, not affecting the isolated influence of the microstructure representation, analyzed in this study. Due to a steeper increase in ventricular pressure compared to the active tension, during isovolumetric contraction, a non-physiological change in volume has been observed. The pressure trace was generated based on subject-specific *in-vivo* measurements and is inconsistent to the cosine-shape active tension resulting from the active model of the living heart framework. The strong hemodynamic constraint of a fixed pressure trace leads to a challenging, if even possible, active parameter fitting problem. An alternative, would be a lumped-parameter model, however, resulting in an idealized pressure trace not directly representing the measured pressure. Another data-driven alternative would be to incorporate the active parameter as Lagrange multipliers in the model implementation, while including both, pressure and volume measurements as model inputs (Asner et al., 2016; Miller et al., 2021). This option is, however, not part of the Living Heart Human model. Therefore, the results during isovolumetric contraction were not evaluated in this study and physiological parameters were calculated from the volume corresponding to the EDP and not from that corresponding to maximal volume.

The twist, given by the relative rotation of apex and base, was evaluated and follows a physiological behaviour. Nevertheless, a remaining net counter-clockwise rotation of the left ventricle was observed at the end of the simulation of one cardiac cycle, related to the missing constraint of the moments. Constraining the average moments of the base to zero on the contrary would prevent basal rotation and thus affect twist. A potential way to compensate the net rotation would be to constrain the average moments to the average moments of the data (Asner et al., 2017).

Linear tetrahedral elements were used in this study to simulate the nearly-incompressible material. Despite the small element size counteracting potential inaccuracies, this might introduce locking and thus an artificial stiffening and torsional rigidity (Maas et al., 2016). Therefore, a potential way to mitigate the underestimation of twist for all compared models could be the use of quadratic tetrahedral elements.

## 6 Conclusion

We have demonstrated that cardiac simulations based on *in-vivo* fiber orientation obtained from cDTI can be performed. We outlined four methods to include patient-specific, *in-vivo* microstructure in a cardiac computational model. The same trend in simulation outputs for all parameter settings was observed and found to coincide with the interpolation precision and accuracy. A decrease in interpolation error was correlated with more physiological twist and higher material stiffness. This suggests that errors in the fiber representation propagate to the simulation results and introduce a systematic bias in the model outcome. To reduce the added fiber uncertainty by the interpolation method, and thereby the bias of the simulation result, the tensor interpolation method (HFC) is the best choice, despite the remaining bias due to the data uncertainty.

## Data Availability

The data analyzed in this study is subject to the following licenses/restrictions: The data was collected from previous publications and is available upon reasonable request to the corresponding author. Requests to access these datasets should be directed to stoeck@biomed.ee.ethz.ch.

## Supplementary material

Supplementary Figure S1 Pressure trace of the forward simulation during one cardiac cycle, starting at the initial diastatic state. This pressure corresponds to the loading condition on the endocardial surface applied upon linear inflation.

Supplementary Figure S2 Personalized model outputs for the HFC-model (with Setting 1) in red, PGD-model (with Setting 2) in violet, POD-model (with Setting 3) in blue, and RBM-model (with Setting 4) in green. **(A)** shows the circumferential, radial, longitudinal, and fiber strain distributions (similar to Figure 7), **(B)** shows the pV-loops, with a marker indicating the initial state (similar to Figure 5), and **(C)** shows the twist over the cardiac cycle. The vertical black line in **(C)** marks the end-systolic time-point where peak twist is evaluated (similar to Figure 8).
