# Erratum: Personalization of biomechanical simulations of the left ventricle by *in-vivo* cardiac DTI data: Impact of fiber interpolation methods

**DOI:** 10.3389/fphys.2023.1171201

**Published:** 2023-03-03

**Authors:** Frontiers Production Office

**Affiliations:** Frontiers Media SA, Lausanne, Switzerland

**Keywords:** *in vivo* cDTI, patient-specific modelling, cardiac microstructure, fiber interpolation, cardiac simualtion, *in vivo* microstructure, personalized modelling

An omission to the **Funding** section of the original article was made in error. The following sentence has been added: “Open access funding was provided by ETH Zurich.”

The original version of this article has been updated.

