# Supplementary figures and images for: Personalization of biomechanical simulations of the left ventricle by in-vivo cardiac DTI data: Impact of fiber interpolation methods
Source: Front Physiol. 2022 Nov 28;13:1042537. doi: 10.3389/fphys.2022.1042537 (PMC9742433; doi:10.3389/fphys.2022.1042537)

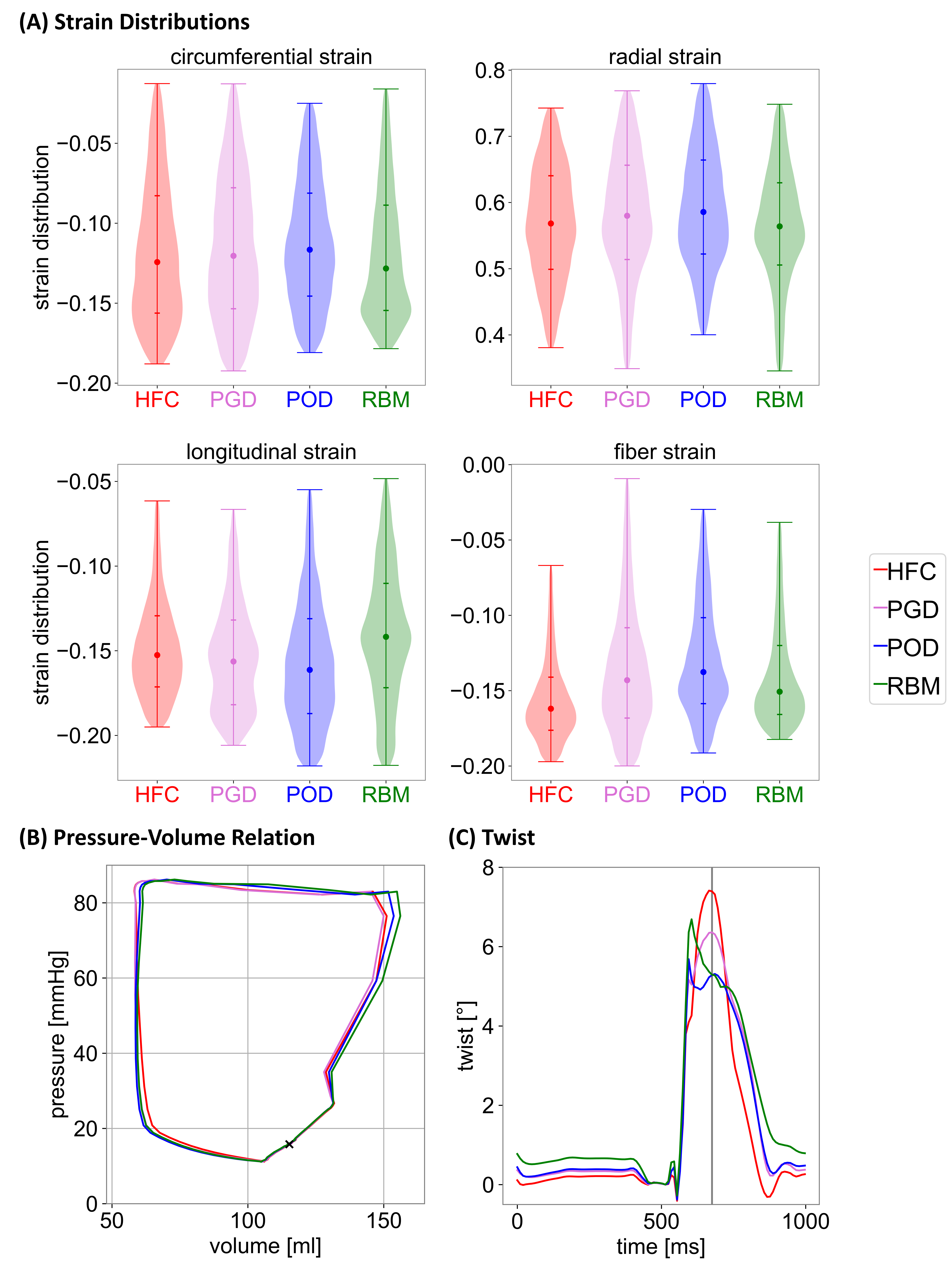

Supplement: Supplementary file 1 [file Image2.PNG]

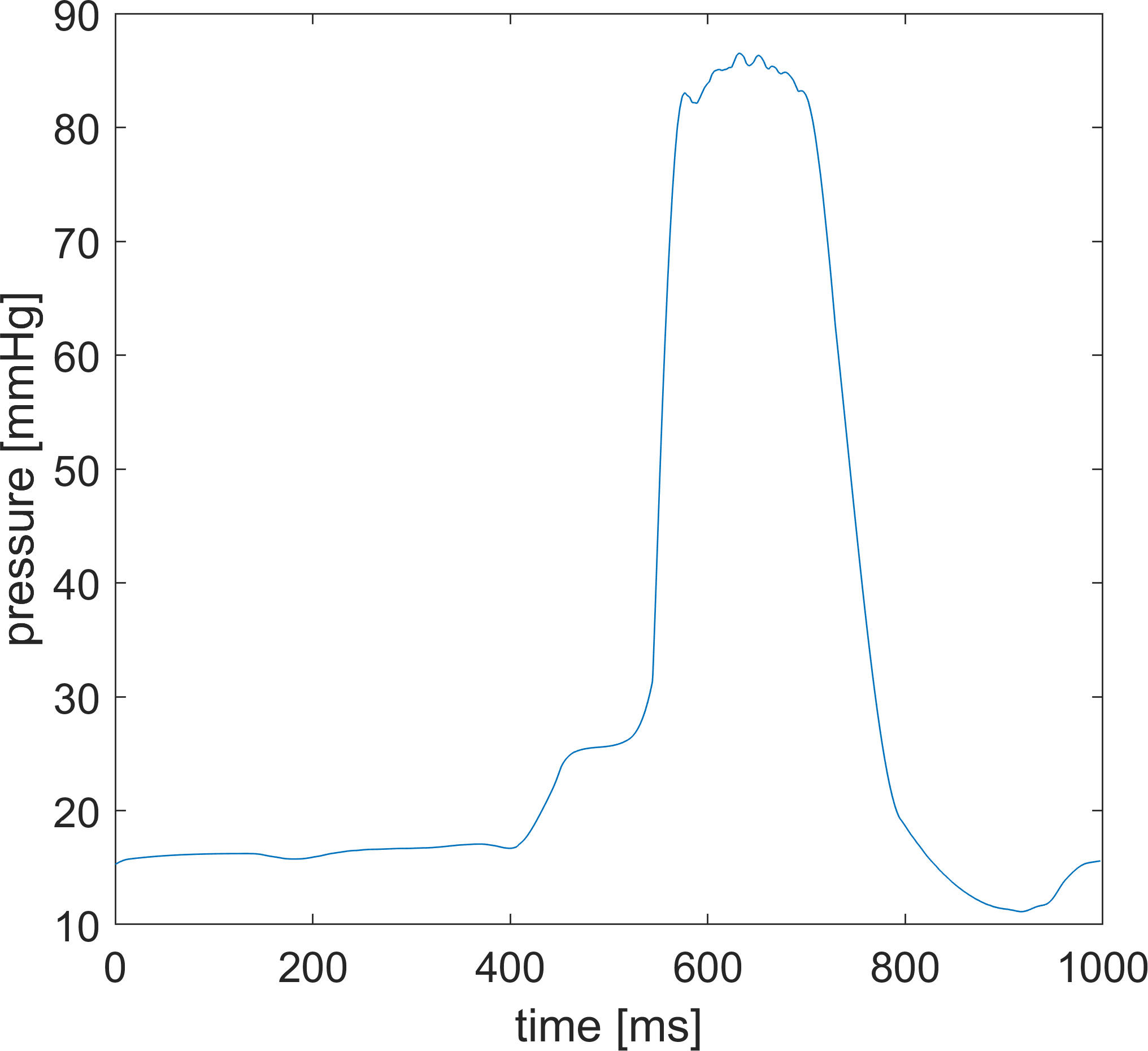

Supplement: Supplementary file 2 [file Image1.PNG]
